# Molecular Alterations in Osteosarcomas of the Oral and Maxillofacial Region: A Scoping Review

**DOI:** 10.1111/jop.70103

**Published:** 2025-12-12

**Authors:** Iara Vieira Ferreira, Reydson Alcides de Lima‐Souza, Moisés Willian Aparecido Gonçalves, Carlos Takahiro Chone, Arthur Antolini‐Tavares, Albina Altemani, Fernanda Viviane Mariano

**Affiliations:** ^1^ Oral Diagnosis Department, Piracicaba Dental School State University of Campinas (UNICAMP) Piracicaba São Paulo Brazil; ^2^ Pathology Department, School of Medical Sciences State University of Campinas (UNICAMP) Campinas São Paulo Brazil; ^3^ Department of Ophthalmology and Otorhinolaryngology, School of Medical Sciences State University of Campinas (UNICAMP) Campinas São Paulo Brazil

**Keywords:** jaw neoplasms, molecular analysis, osteosarcoma, scoping review

## Abstract

**Background:**

Given the rarity and aggressive nature of osteosarcomas (OS) in the oral and maxillofacial region, understanding their molecular alterations is essential to improve diagnosis, prognosis, and guide targeted therapies. This study aimed to map molecular alterations associated with oral and maxillofacial OS, providing an overview of the genetic mechanisms involved in their development and progression.

**Methods:**

A scoping review was performed following the PRISMA‐ScR guidelines. Studies included observational research, case reports, and systematic reviews focusing on molecular alterations in oral and maxillofacial OS. A comprehensive search was conducted across four databases, and findings were synthesized and categorized by molecular characteristics.

**Results:**

A total of 20 studies involving 68 maxillofacial OS cases were included. The average patient age was 39.4 years, with a slight male predominance. The mandible was the most commonly affected site, and chondroblastic OS was the most frequent histological subtype. Genetic alterations were predominantly observed in the *TP53* gene, along with alterations in *MDM2*, *CDK4*, and other genes. Treatment primarily involved surgery, with or without chemotherapy. Local recurrence occurred in 11.1% of cases, and distant metastases in 16%. At the final follow‐up, 69.7% of patients were alive.

**Conclusion:**

This study emphasizes the value of molecular techniques in improving the diagnosis and management of maxillofacial OS. However, further research is needed to fully understand the molecular complexity and optimize therapeutic strategies.

## 1 Introduction

Osteosarcoma (OS) is a rare malignant bone neoplasm with a global incidence of approximately 4–5 cases per million people per year. It is characterized by the production of immature bone by tumor [1]. While the femur, tibia, and humerus are the most common sites, the jaws—particularly the mandible—represent the fourth most common site [2]. In contrast to OS affecting the long bones of the extremities, which display a bimodal incidence pattern with most cases occurring between 14 and 18 years and a smaller second peak in older adults, OS in the head and neck region typically develops in patients 10 to 20 years older than those with extragnathic OS, predominantly in the third to fourth decades of life, with no sex predilection [1, 3]. This neoplasm can present as a primary tumor, associated with genetic factors such as Li‐Fraumeni, Werner, Bloom, Rothmund‐Thomson, and retinoblastoma syndromes, associated with mutations in *TP53*, *RECQL2*, *BLM*, *RECQL4*, and *RB1*, respectively. Additionally, it can occur as a secondary tumor, often related to previous radiation therapy, a history of retinoblastoma, or Paget's disease [1, 4].

Craniofacial OS differs from axial skeleton OS in its distinct clinical and biological behavior [5]. In the craniofacial region, OS exhibits a reduced metastasis rate compared to cases originating from extracranial regions. Nevertheless, the recurrence rate remains elevated, primarily due to the challenges associated with achieving complete resection in anatomically complex areas [5, 6]. The standard treatment remains complete surgical resection, though the role of neoadjuvant chemotherapy continues to be a topic of debate [5, 7]. Although progress has been made in the treatment of OS in long bones, its rare occurrence in the head and neck region hinders a comprehensive understanding of its behavior in this area, contributing to persistently unsatisfactory therapeutic outcomes due to its pronounced biological aggressiveness [8, 9].

In recent years, advances in the study of molecular alterations have allowed better stratification of lesions, aiding in diagnosis, prognostic prediction, and the development of targeted therapies [10]. Low‐grade OS often shows gene amplification in *CDK4* and *MDM2* located on chromosome 12q13–15. *MDM2* amplification is highly specific and distinguishes these tumors from benign fibro‐osseous lesions [2, 11]. In contrast, high‐grade OS are characterized by complex karyotypes with numerous structural and numerical abnormalities, often associated with chromothripsis [12].

A deeper understanding of the molecular mechanisms underlying the clinical behavior and treatment resistance of these tumors is essential to improve patient outcomes. In this context, the present study aims to map the molecular alterations associated with prognosis in craniofacial OS, providing a basis for the development of targeted therapeutic strategies. The guiding question of this scoping review is: “What molecular alterations have been described in the literature for OS affecting the oral and maxillofacial region?”

## 2 Material and Methods

### 2.1 Protocol and Registration

The methods of this scoping review were previously established according to the Preferred Reporting Items for Systematic Reviews and Meta‐Analyses for Systematic Review Protocols (PRISMA‐P) checklist [13]. The resulting protocol was registered with the Open Science Framework (OSF; Center for Open Science, Charlottesville, United States) (Spies JR, [14]), and is available online with the registration number DOI 10.17605/OSF.IO/W35KZ. In addition, the present scoping review was conducted following the checklist of the PRISMA Extension for Scoping Reviews (PRISMA‐ScR) [15, 16].

### 2.2 Eligibility Criteria

The acronym PCC (Population, Concept, Context) was used to formulate the review question and establish the inclusion criteria. For this review, the defined criteria were as follows: (P) Oral and maxillofacial OS; (C) Molecular alterations; (C) Prognostic and diagnostic implications. Eligible for inclusion were observational studies (cohort studies, case–control studies, or cross‐sectional studies), case reports, as well as systematic reviews published in English, Spanish, and Portuguese.

The exclusion criteria were as follows: (1) Studies that did not address molecular alterations of oral and maxillofacial OS; (2) Clinical trials, experimental studies, narrative reviews, protocols, brief communications, personal opinions, letters, book chapters, and conference abstracts; (3) Studies for which full‐text articles were not available; (4) Studies published in languages other than English, Spanish, or Portuguese; (5) Insufficient clinicopathological data.

### 2.3 Information Sources and Search Strategy

The search strategies were conducted on January 18, 2025, without restrictions on publication date, and were developed by combining appropriate keywords and index terms to find all relevant studies addressing the review question. Bibliographic databases, including MEDLINE (PubMed), Scopus, Web of Science, and EMBASE, were used to identify articles on this topic. A gray literature search, including Google Scholar and ProQuest Dissertation and Theses, was also carried out to identify unpublished references. Additionally, the reference lists of included studies were cross‐checked. The search strategy is detailed in Table S1. The retrieved studies were imported into the reference manager Rayyan [17], where duplicate references were removed.

### 2.4 Selection of Sources of Evidence

The study selection process was performed in two phases by two independent reviewers (IVF and RALS). In the first phase, the titles and abstracts of the studies selected in Rayyan [17] were reviewed. Studies that met all inclusion criteria proceeded to the second phase, which involved a full‐text review and confirmation of eligibility criteria. In case of disagreement or uncertainty between the two reviewers, the third reviewer (FVM) was consulted to resolve the issue through a consensus meeting.

### 2.5 Data Charting Process and Data Items

The data from the included studies were collected by one reviewer (IVF) and reviewed by a second reviewer (RALS). Disagreements were resolved by discussion and consensus among the authors. The extracted data were organized and processed in Microsoft Excel. The main data extracted included: study and population characteristics, molecular alterations, and prognostic information.

### 2.6 Synthesis of Results

A qualitative synthesis was performed by grouping the data from all included studies according to similar features to determine frequency data for each of the characteristics of interest and summarizing the type of settings for the category of interest, describing settings, measures, and general outcomes.

## 3 Results

### 3.1 Selection of Sources of Evidence

Of the 2265 records identified through database searches, 1488 remained after duplicate removal. These records were then screened for eligibility using a two‐phase selection process. In the first phase, titles and abstracts were reviewed, resulting in 95 studies selected for full‐text analysis in the second phase. Following this assessment, 17 studies were included in the descriptive synthesis. Additionally, 3 studies were identified through other methods, including gray literature and manual reference checking of the selected studies, bringing the total to 20 studies [9, 18, 19, 20, 21, 22, 23, 24, 25, 26, 27, 28, 29, 30, 31, 32, 33, 34, 35, 36] (Figure S1), The reasons for exclusion in the second phase 2 are detailed in Table S2.

### 3.2 Characteristics of Sources of Evidence

The detailed information of each study is available in Table S3. The studies were conducted in the following countries: the United States (5), Japan (3), France (3), Brazil (1), Australia (1), Canada (1), China (1), India (1), Iran (1), Spain (1), the United Kingdom (1), and Switzerland (1) (Figure S2). Among them, 13 were case reports, 3 were cohort studies, and 4 were cross‐sectional studies. All studies were published in English between 1990 and 2024, with the highest number of publications in 2024 (5 studies).

### 3.3 Clinical and Demographic Characteristics

A total of 68 patients diagnosed with OS in the maxillofacial region were analyzed. Among them, one patient developed a second primary tumor 2 years after the diagnosis of the first primary tumor [32]. The summarized data are presented in Table 1.

**TABLE 1:** Summary of 68 oral and maxillofacial osteosarcomas.

Clinicopathological variables	Value %
Sex (*n* = 55)	
Male	28 (50.9%)
Female	27 (49.1%)
Age (years) (*n* = 66)	
Mean	39.39 ± 20.4
Median	38.5
Range	6–81
Tumor location (*n* = 66)	
Mandible	46 (69.7%)
Maxilla	18 (27.3%)
Sphenoid	1 (1.5%)
Frontal sinus and infratemporal fossa	1 (1.5%)
Size (cm) (*n* = 30)	
Mean	4.8 ± 2.4
Median	4.35
Range	1.5–11
Histologic subtype (*n* = 56)	
Chondroblastic	28 (50%)
Osteoblastic	22 (39.2%)
Fibroblastic	18 (32.1%)
Telangiectatic	2 (3.5%)
Giant cell‐rich	1 (1.7%)
Parosteal	1 (1.7%)
Grade (*n* = 58)	
High	46 (79.3%)
Intermediate	5 (8.6%)
Low	7 (12.1%)
Genetic factor: Li‐Fraumeni syndrome (*n* = 32)	
No	17 (53.1%)
Yes	15 (46.9%)
Risk factor (*n* = 15)	
No	7 (46.7%)
History of radiation exposure in the region	8 (53.3%)
Treatment (*n* = 30)	
Surgery	27 (90%)
Chemotherapy	17 (56.6%)
Radiotherapy	4 (13.3%)
Margin status (*n* = 12)	
Negative	11 (91.6%)
Positive	1 (8.4%)
Local recurrence (*n* = 27)	
No	24 (88.8%)
Yes	3 (11.1%)
Metastasis (*n* = 25)	
No	21 (84%)
Yes	4 (16%)
Status (*n* = 33)	
Alive	23 (69.7%)
Died	8 (24.2%)
Lost follow‐up	2 (6.1%)
Time of follow‐up (months) (*n* = 31)	
Mean	23.6 ± 24.1
Median	16
Range	2–120

Out of the 55 patients with available sex information, the majority were male, representing 50.9% of the total. The age range spanned from 6 to 81 years, with a mean age of 39.3 ± 20.4 years and a median age of 38.5 years. Regarding tumor location, the mandible was the most frequently affected site, involving 46 patients (69.7%), followed by the maxilla, which was affected in 18 patients (27.3%). For tumor size, 30 cases provided this information, and the average largest diameter was 4.8 ± 2.4 cm, with a range from 1.5 cm to 11 cm.

### 3.4 Histopathological Features and Staging

In terms of histological subtype, data from 56 cases were available. Among these, the majority were classified as chondroblastic (28 cases, 50%), followed by osteoblastic (22 cases, 39.2%), and fibroblastic (18 cases, 32.1%). Some tumors were reported with more than one histological subtype per lesion, including 6 cases (10.7%) classified as both osteoblastic and chondroblastic, and 5 cases (8.9%) as osteoblastic, chondroblastic, and fibroblastic. Additionally, two cases (3.5%) were classified as telangiectatic [25, 33], one case (1.7%) as giant cell‐rich [24], and one case (1.7%) as parosteal [9].

Among the 58 patients with available histological grade data, the majority of tumors (46 cases, 79.3%) were classified as high‐grade. Lymphovascular invasion was documented in only one case [33]. Data on neural or perineural invasion were not reported, and information on necrosis was available for four cases, with three exhibiting necrosis [23, 25, 29]. Additionally, data regarding the stage were provided for only one case, which was classified as stage IA (pT1 and pN0) [33].

### 3.5 Risk Factors and Genetic Predispositions

Regarding genetic factors, data from 32 patients were available. Among them, 15 patients (46.9%) were diagnosed with Li‐Fraumeni syndrome (LFS) [18, 21, 23, 27, 30, 31, 32]. Among these, eight patients had a documented history of additional cancers. Of these, five had a prior history of OS. The remaining three patients had the following medical histories: one with disorders of sex development, OS, and Bowen's disease; another with lobular breast carcinoma; and one with multiple malignancies, including neuroblastoma, rhabdomyosarcoma, phyllodes tumor, bronchioloalveolar carcinoma, invasive ductal breast carcinoma, papillary thyroid carcinoma, OS, gastric adenocarcinoma, and acute myeloid leukemia.

In terms of risk factors, data for 15 patients revealed that eight (53.3%) had a history of radiation exposure in the affected region [9, 21, 22, 27, 35, 36]. Among these 8 patients, 3 had tumors related to postradiotherapy effects: 1 with cerebral astrocytoma, 1 with papillary thyroid carcinoma (latency period of 11 years), and 1 with squamous cell carcinoma (latency of 13 years, total dose of 60 Gy). Although not considered a risk factor for OS by the World Health Organization (WHO) [1], one patient (1.4%) had cemento‐osseous dysplasia [23], and two (2.9%) had fibrous dysplasia [34, 36].

### 3.6 Molecular Profiles

In the 68 tumors analyzed, a variety of molecular tests were employed to investigate genetic alterations, as detailed in Table S3. The *TP53* gene was the most frequently altered. Amplification was observed in 4 out of 32 cases, while missense mutations occurred in 23 cases, frameshift mutations in 2 cases, in‐frame deletion in 1 case, and a splice variant in 1 case. Additionally, for 1 case, it was unclear whether the mutation was a missense or a frameshift mutation. *MDM2* amplification was found in 13 out of 30 cases, while *CDK4* amplification was observed in 7 out of 12 tested cases. *SAS* (Sarcoma Amplified Sequence) gene amplification was noted in 6 out of 9 cases, and *RASAL1* amplification was detected in 3 out of 14 tested cases. Additionally, three missense mutations in *GNAS* were identified: R201C, R201H, and A201C.

Reinforcing the molecular complexity of OS, various genetic alterations were observed, although less frequently. Among the amplifications, the genes *CCNE1*, *KEL*, *EZH2*, *XRCC2*, *PTEN*, *TERT*, *C17orf39*, and *KRAS* stand out. Missense and truncation mutations were identified in genes such as *MUC4*, *MUC6*, *MUC17*, *MUC20*, *HLA*, *ZNF221*, *ZNF417*, *ZNF517*, *ZNF595*, *ZNF774*, *ZNF831*, *CYP2A7*, *CYP27C1*, *GADD45B*, *MSH4*, *TDG*, *MCM4*, *RBBP8NL*, *PER3*, *CDK11B*, *CDC27*, *CCNE1*, *LTK (S183F)*, *BRCA2 (S758C)*, *ERBB3 (R1118Q)*, *KMT2A (MLL) (G73E)*, *KMT2C*, *APC*, *RAD50*, *PTEN*, *RB1*, *LTK (W707*), and *PBRM1*. Additionally, in‐frame fusion was detected in *RAD50*, in‐frame deletion in *RB1*, and a splicing site mutation in *RB1* (*539 + 1G>A*). Homozygous deletions were identified in *CDKN2A/2B*, *PTEN*, and *RB1*, while a hotspot mutation in the promoter was found in *TERT*. Chromoplexy events led to subsequent amplifications on 5p, 8pter, 12, and 19p, as well as deletions on 5q, 6, 7, 10p, 13, and 22. In one of the cases analyzed, an aberrant transcript of the *WWOX* gene was identified, showing total or partial exon loss.

### 3.7 Treatment, Tumor Behavior, and Follow‐Up

Out of the 30 patients with available treatment information, surgery was the most common treatment modality, performed in 27 patients (90%). Among them, 14 (51.9%) underwent surgery combined with chemotherapy, 11 (40.7%) had surgery alone, 1 (3.7%) received surgery with radiotherapy and chemotherapy, and 1 (3.7%) underwent surgery with radiotherapy. Additionally, 3 patients (10%) were treated with radiotherapy alone (1; 3.3%), chemotherapy alone (1; 3.3%), or a combination of both treatments (1; 3.3%).

Regarding surgical margins, data from 12 cases were available. In 11 cases (91.6%), the margins were negative, though two were close. One case (8.4%) showed a positive margin. Local recurrence, assessed in 27 cases, was observed in three patients (11.1%), while 24 patients (88.8%) remained recurrence‐free. Distant metastases, evaluated in 25 cases, occurred in four patients (16%), affecting the liver, lungs, bone marrow, and multiple bone sites. In one case, it was unclear whether the sternal spindle cell sarcoma was a primary tumor or a metastasis from maxillary OS. Follow‐up data were available for 31 cases, with a mean duration of 23.66 ± 24.18 months, ranging from 2 to 120 months. At the last follow‐up, 23 patients (69.7%) were alive, while 8 (24.2%) had died.

### 3.8 Association Between Molecular Alterations and Prognosis

Of the 19 patients with *TP53* gene alteration, 11 had high‐grade tumors. Of these, 3 experienced local recurrence, and 4 developed distant metastases. After a mean follow‐up of 28.3 ± 27.8 months, 12 out of 19 remained alive. Among the 7 patients with *MDM2* amplification, 4 had high‐grade tumors. None showed local recurrence or metastases. With a mean follow‐up of 15 ± 9.9 months, 6 out of 7 were still alive.

In the group of 6 patients with *CDK4* gene amplification, 3 cases were classified as high‐grade tumors. Similar to the previous group, no local recurrence or metastases were observed. After a mean follow‐up of 15 ± 10.6 months, all patients remained alive. Lastly, among the six patients with SAS amplification, half had low‐grade tumors. None of these patients developed local recurrence or metastases. With a mean follow‐up of 16.8 ± 9.7 months, all patients were alive at the time of the analysis.

## 4 Discussion

OS in the maxillofacial region presents significant differences from OS in long bones, both in clinical presentation and in biological behavior and prognosis. In the oral and maxillofacial region, OS primarily affects the mandible and maxilla, with common symptoms such as swelling, pain, dental mobility, and paresthesia [36, 37]. In this study, 97% of the cases involved these sites. Radiologically, maxillofacial OS is highly aggressive, characterized by destructive growth, periosteal reaction, and extension into soft tissues. Computed tomography is the imaging modality of choice for staging and surgical planning, while magnetic resonance imaging (MRI) is essential for assessing intraosseous extension and soft tissue involvement [2, 38].

According to the current WHO classification [1], OS in the gnathic region includes the following subtypes: conventional, small cell, telangiectatic, central low‐grade, parosteal, periosteal, high‐grade surface, giant cell‐rich, and radiation‐induced. The conventional subtype is the most common and aggressive, while the periosteal subtype is of intermediate grade, and the central and parosteal subtypes are classified as low‐grade. Conventional OS, characterized as a high‐grade intraosseous tumor, presents highly atypical cells, with the main diagnostic feature being the production of osteoid tumor. Its wide morphological variation allows subclassification into three main types, based on the predominant matrix formed: osteoblastic, chondroblastic, and fibroblastic [2, 39]. Rarer subtypes, such as small cell and telangiectatic OS, are extremely uncommon in the gnathic region [39]. In our study, we observed one case of telangiectatic OS, one case rich in giant cells, and one case of parosteal OS. The giant cell‐rich OS is a morphological subtype that should be remembered and differentiated from other giant cell‐rich lesions affecting the jaws [1]. The majority of the cases were chondroblastic (50%) and classified as high‐grade (79.3%).

Although most cases of OS are sporadic, there are recognized risk factors and genetic predispositions associated with its development. Known predisposing factors include Paget's disease, a history of retinoblastoma, and prior radiation exposure [40]. Additionally, patients with LFS, who carry germline mutations in the *TP53* gene, have a higher incidence of OS [40]. LFS is associated with a high rate of multiple tumors, especially metachronous ones [32]. In our study, 15 patients (46.9%) were diagnosed with LFS, and 8 of them had a history of additional cancers. All 15 cases were positive for the *TP53* mutation. Early diagnosis is crucial for implementing appropriate strategies and adjusting follow‐up based on the risk of developing a second cancer. Without systematic germline *TP53* mutation testing, identifying LFS in OS patients can be challenging, especially when it is the first malignancy [32, 41].

Radiation‐associated maxillofacial OS is rare but represents the most common radiation‐related sarcoma subtype in the head and neck region. The latency period following radiation is, on average, 11 years, ranging from 4 to 23 years [40]. In our study, 8/15 (53.3%) patients had a history of radiation exposure in the region. Compared to primary maxillofacial OS, radiation‐induced OS tends to be more aggressive, high‐grade, and fibroblastic, with elevated p53 protein expression levels, as well as a less favorable prognosis [40, 42]. *TP53* mutations, associated with p53 overexpression, play a crucial role in the pathogenesis of radiation‐induced OS. Radiation‐associated maxillofacial OS exhibits a higher frequency of mutations and p53 overexpression compared to primary tumors. However, most studies suggest that p53 expression has no significant prognostic value [9, 42]. In our study, of the 8 cases of radiation‐induced OS, 4 were classified as high‐grade, and 3 were positive for *TP53*.

The differentiation between low‐grade OS and benign fibro‐osseous lesions of the maxillofacial region, such as fibrous dysplasia and ossifying fibromas, can be challenging, especially in the absence of comprehensive imaging or when biopsy material is limited [39]. Molecular testing plays a crucial role in achieving a more accurate diagnosis. From a cytogenetic perspective, low‐grade OS frequently presents recurrent alterations, such as ring chromosomes containing multiple copies of the 12q13‐15 region, which harbors the *MDM2* and *CDK4* genes [39, 43]. *MDM2* gene amplification has been observed in over 60% of low‐grade OS, while it is absent in benign fibro‐osseous lesions [44]. Interestingly, however, amplification of *MDM2* and *RASAL1* has been reported in a subset of juvenile trabecular ossifying fibromas (7 of 10 cases) [45]. In our study, *MDM2* was positive in 13 cases, with 10 classified as high‐grade, while *CDK4* alterations were identified in 7 cases, including 4 of high‐grade.

The GNAS gene mutation is present in 50%–80% fibrous dysplasias, being a widely recognized marker of this condition. Although rare, it has also been found in low‐grade OS [46] and other fibro‐osseous lesions [43]. In our study, the GNAS gene mutation was identified in three high‐grade OS cases [34, 36]. Zhu et al. [36] reported two cases of high‐grade OS, one of which had a history of fibrous dysplasia. Both cases presented concurrent mutations in the *TP53* and *APC* genes, suggesting a possible synergistic effect in the development of OS. Similarly, [34] described a case of high‐grade OS associated with fibrous dysplasia, proposing that *GNAS* and *TP53* mutations may play a role in the transformation of fibrous dysplasia into OS. These findings highlight the importance of interpreting molecular test results with caution and emphasize the need to integrate molecular data with clinical‐pathological findings to improve diagnostic accuracy [46].

A diagnostic dilemma also arises when differentiating chondrosarcoma from the chondroblastic variant of OS, especially because the management of each tumor is distinct [2]. The chondroblastic variant, which is common in the mandible, can mimic chondrosarcoma, which is rare at this site. The presence of tumoral osteoid, characteristic of OS, is a pathognomonic marker that allows for the distinction between the chondroblastic OS and chondrosarcoma [39]. Furthermore, molecular tests may be useful to confirm the diagnosis, as 49%–61% of chondrosarcomas harbor mutations in *IDH1/2*. Detection of these mutations supports the diagnosis of chondrosarcoma. Consistent with the literature, in our study, no cases of OS presented mutations in *IDH1/2*. However, since not all chondrosarcomas have *IDH1/2* mutations, the wild‐type form of the gene cannot be used to completely exclude a diagnosis of chondrosarcoma [47].

**FIGURE 1:**
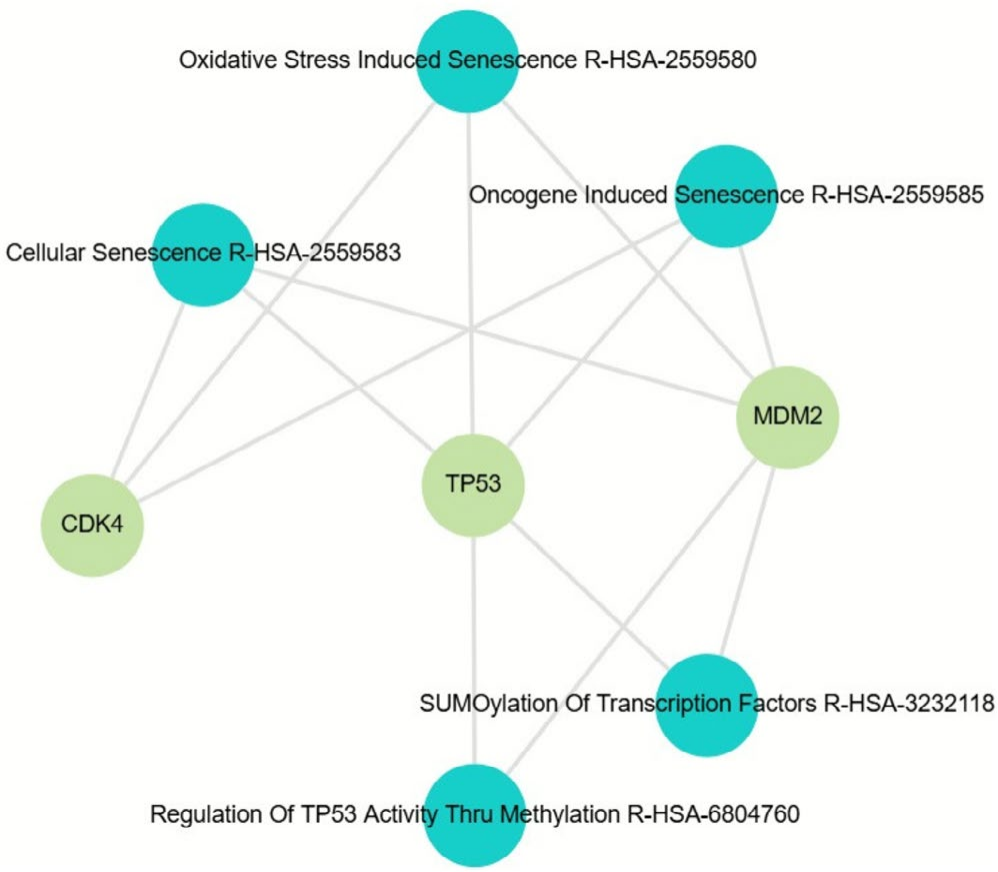
Network analysis of the most frequently altered genes in OS, including *TP53*, *MDM2*, *CDK4*, *SAS*, *RASAL1*, and *GNAS*. Only *TP53*, *MDM2*, and *CDK4* showed statistically significant associations with key biological pathways: Oxidative stress‐induced senescence (*p* = 1.93e‐6), oncogene‐induced senescence (*p* = 8.16e‐8), cellular senescence (*p* = 1.08e‐5), SUMOylation of transcription factors (*p* = 1.28e‐5), and regulation of *TP53* activity through methylation (*p* = 1.28e‐5). Green nodes represent genes, while blue nodes indicate the associated Reactome pathways.

The molecular landscape of OS is still not fully understood. Although few recurrent mutations have been identified, *TP53* and *RB1* are among the most frequently altered genes, contributing to the genomic instability characteristic of this tumor [1]. In our review, alterations were observed in 46 genes, reflecting the molecular complexity of OS. *TP53* was the most frequently altered gene, followed by *MDM2*, *CDK4*, *SAS*, *RASAL1*, and *GNAS*, while *RB1* was described in only one case. *TP53*, known as the “guardian of the genome,” regulates cell cycle arrest, DNA repair, and apoptosis, and its mutation compromises these processes, favoring tumor proliferation. *RB1* controls the G1‐to‐S phase transition, and its inactivation leads to deregulated cell replication [48]. *CDK4* phosphorylates *RB1*, promoting cell cycle progression, and its overexpression can accelerate proliferation, while *MDM2*, by inhibiting *TP53*, reduces the cellular stress response, facilitating tumor development [44, 49]. *SAS* is involved in cell proliferation, *RASAL1* regulates the RAS signaling pathway, contributing to tumor growth, and *GNAS* acts in G‐protein signaling, influencing cell proliferation and differentiation, so that alterations in these genes favor tumor progression [9, 22, 34] (Figure 1).

Genetic data on OS of the gnathic bones is scarce, and it is still unclear whether these tumors share the same pathophysiology as extragnathic OS. To date, no comprehensive genomic analysis has been conducted specifically on gnathic bone OS [36]. This scarcity of molecular data, along with the particularities of handling bone tissue, should be taken into account when planning molecular studies, as decalcification required for paraffin embedding and immunohistochemistry can compromise DNA and RNA. In general, OS exhibits a highly complex genomic landscape, characterized by multiple chromosomal rearrangements and chromothripsis events, as well as aneuploid karyotypes and numerous structural and numerical aberrations, reflecting significant genomic instability [50]. High‐grade OS has an even more complex genomic profile, with numerous aberrations, possibly resulting from cataclysmic events such as chromothripsis or chromoplexy [2, 12]. Although the trigger for these disruptive chromosomal alterations is not yet fully understood, *TP53* inactivation appears to play a crucial role [2]. Unlike low‐grade OS, high‐grade OS lacks a specific molecular signature, making their diagnosis and characterization even more challenging [43].

Gnathic bone OS has a more favorable prognosis compared to long bone OS, with higher survival rates and a lower frequency of metastases, which typically occur at more advanced stages of the disease [36]. However, local disease control remains challenging, often leading to treatment failures [37]. Surgical resection with clear margins is considered the standard treatment. The role of adjuvant therapy in oral and maxillofacial OS remains controversial, although studies suggest that chemotherapy may improve survival outcomes [51]. In this study, positive surgical margins were found in 1/12 patients (8.4%), local recurrence occurred in 3/27 cases (11.1%), and distant metastases developed in 4/25 patients (16%). Surgery was the primary treatment modality, performed in 27/30 cases (90%), either with chemotherapy in 14/27 (51.9%) or alone in 11/27 (40.7%). After a mean follow‐up of 23.6 months, 23/33 patients (69.7%) were alive. These findings align with the literature, which identifies positive resection margins as a strong predictor of poor prognosis and highlights the potential benefit of chemotherapy in improving survival for patients with high‐grade tumors, local recurrence, or positive margins [52]. Notably, radiation‐induced OS presents a significantly worse prognosis, with higher morbidity and mortality rates compared to primary OS [40].

Immunohistochemistry is a widely accessible and commonly used ancillary tool in diagnostic practice. However, unlike OS from other anatomical sites, few studies have specifically evaluated its performance in the oral and maxillofacial region. As a result, the sensitivity, specificity, and overall diagnostic reliability of these markers in gnathic OS remain uncertain. According to the current WHO classification of head and neck tumors, positive staining for MDM2 and CDK4 may help distinguish low‐grade osteosarcoma from benign fibro‐osseous lesions [53, 54], while SATB2 can indicate osteoblastic differentiation, although its lack of specificity limits its diagnostic value despite relatively high sensitivity [55, 56]. Notably, most studies supporting these recommendations were conducted on OS from other anatomical sites, with very limited data available specifically for tumors of the oral and maxillofacial region. In this context, p53 expression may provide additional diagnostic insight, as superexpression of *TP53* is frequently observed in high‐grade OS [42]; however, this finding alone is insufficient to distinguish these tumors from mimicking lesions, since some benign lesions or low‐grade tumors may exhibit weak or focal positivity.

The present study identified a large number of altered genes, highlighting the molecular complexity of OS and providing new perspectives for future studies. However, some limitations should be considered, such as the sample size, which may not fully capture the genetic heterogeneity of OS, and the absence of a clear correlation between genetic alterations and patient clinical outcomes. The genetic mapping of these lesions is crucial to identify prognostic correlations, guiding therapeutic decisions, and improving patient monitoring over time. Therefore, additional studies are needed to validate these findings, explore their clinical relevance, and investigate their application in personalized treatment strategies, especially in gnathic OS. In this context, conducting a systematic review on the subject is warranted, as it would allow a comprehensive synthesis of the available evidence, the appraisal of study quality, and the identification of knowledge gaps that may guide future research. This is justified by the fact that OS in the maxillofacial region presents significant differences compared to OS in long bones, both in clinical presentation and biological behavior and prognosis, with studies focusing on this specific region still being scarce.

## 5 Conclusion

Analysis of 68 cases of oral and maxillofacial OS revealed a predominance of aggressive and high‐grade forms, with the mandible being the most affected site. Most cases exhibited histologic features typical of conventional OS, with a predominance of the chondroblastic and osteoblastic subtypes. In total, alterations were observed in 46 genes, reflecting the molecular complexity of OS. *TP53* was the most frequently altered gene, followed by *MDM2*, *CDK4*, *SAS*, *RASAL1*, and *GNAS*. *TP53* mutations disrupt cell cycle control, DNA repair, and apoptosis, while *MDM2* promotes tumor proliferation by inhibiting *TP53*. *CDK4* drives cell cycle progression via *RB1*, *SAS* enhances proliferation, and *RASAL1* and *GNAS* contribute to tumor growth and progression. The study also emphasized the influence of genetic factors, such as mutations in the *TP53* gene and predisposing conditions. Prior radiation exposure was identified as a relevant factor in a significant proportion of patients, highlighting the role of prior treatments in the pathogenesis of oral and maxillofacial OS.

Molecular techniques have proven to be essential in the diagnostic approach, allowing for a more detailed analysis of genetic alterations, facilitating the differentiation of subtypes, and improving case management. The standard treatment primarily involves surgical resection. Despite a relatively high survival rate, local disease control and the risk of recurrence remain significant challenges, highlighting the need for new therapeutic approaches and follow‐up strategies to optimize long‐term prognosis. Further research is essential to deepen our understanding of the molecular complexity of oral and maxillofacial OS and to develop improved therapeutic options, ultimately leading to better outcomes.

## Author Contributions

**Iara Vieira Ferreira:** conceptualization, investigation, writing – original draft, methodology, data curation, formal analysis. **Reydson Alcides de Lima‐Souza:** conceptualization, investigation, writing – original draft, methodology, data curation, formal analysis. **Moisés Willian Aparecido Gonçalves:** data curation, formal analysis, writing – original draft. **Carlos Takahiro Chone:** writing – review and editing. **Arthur Antolini‐Tavares:** writing – review and editing. **Albina Altemani:** writing – review and editing. **Fernanda Viviane Mariano:** conceptualization, supervision, writing – review and editing.

## Funding

This work was supported by Coordenação de Aperfeiçoamento de Pessoal de Nível Superior (001), and Fundação de Amparo à Pesquisa do Estado de São Paulo (20/08431‐4, 23/14770‐4).

## Conflicts of Interest

The authors declare no conflicts of interest.

## Supporting information


**Data S1:** Supporting Information

## Data Availability

The data that supports the findings of this study is available in the Supporting Information S1 of this article.
