# Supplementary material for: Molecular Alterations in Osteosarcomas of the Oral and Maxillofacial Region: A Scoping Review
Source: J Oral Pathol Med. 2025 Dec 12;55(4):439–47. doi: 10.1111/jop.70103 (PMC13065887; doi:10.1111/jop.70103)

**Table S1** Search strategies with appropriate key words and number of references retrieved from each database and grey literature.

| **Database** | (Search date: January 18^th,^ 2025) | **Results** |
| --- | --- | --- |
| **PubMed** | (Osteosarcoma[Mesh] OR Osteosarcomas OR "Osteogenic sarcoma" OR "osteosarcoma tumor" OR "osteosarcoma tumors" OR "Tumor, Osteosarcoma" OR "Tumors, Osteosarcoma" OR "Sarcoma, Osteogenic" OR "Osteogenic Sarcomas" OR "Sarcomas, Osteogenic" OR “bone tumours” OR “bone tumor" OR “Neoplasms, bone tissue"[Mesh]) **AND** ("Gene Expression"[Mesh] OR Mutation[Mesh] OR "Pathology, Molecular"[Mesh] OR "Chromosome Aberrations"[Mesh] OR "molecular diagnostics" OR "molecular markers" OR "molecular alterations" OR "genetic alterations" OR "genomic analysis" OR "molecular profiling") **AND** (Mouth[Mesh] OR Jaw[Mesh] OR Maxillofacial OR "Head and Neck" OR "oral and maxillofacial" OR jaws OR mandible OR maxilla OR craniofacial OR oral OR “oral cavity” OR extraskeletal) | 234 |
| **Scopus** | TITLE-ABS-KEY (Osteosarcoma OR Osteosarcomas OR "Osteogenic sarcoma" OR "osteosarcoma tumor" OR "osteosarcoma tumors" OR "Tumor, Osteosarcoma" OR "Tumors, Osteosarcoma" OR "Sarcoma, Osteogenic" OR "Osteogenic Sarcomas" OR "Sarcomas, Osteogenic" OR “bone tumours” OR “bone tumor" OR “Neoplasms, bone tissue") **AND** TITLE-ABS-KEY ("Gene Expression" OR "Mutation" OR "Pathology, Molecular" OR "Chromosome Aberrations" OR "molecular diagnostics" OR "molecular markers" OR "molecular alterations" OR "genetic alterations" OR "genomic analysis" OR "molecular profiling") **AND** TITLE-ABS-KEY (Mouth OR Jaw OR Maxillofacial OR "Head and Neck" OR "oral and maxillofacial" OR jaws OR mandible OR maxilla OR craniofacial OR oral OR "oral cavity" OR extraskeletal) | 832 |
| **Embase** | (Osteosarcoma OR Osteosarcomas OR 'Osteogenic sarcoma' OR 'osteosarcoma tumor' OR 'osteosarcoma tumors' OR 'Tumor, Osteosarcoma' OR 'Tumors, Osteosarcoma' OR 'Sarcoma, Osteogenic' OR 'Osteogenic Sarcomas' OR 'Sarcomas, Osteogenic' OR 'bone tumours' OR 'bone tumor' OR 'Neoplasms, bone tissue') **AND** ('Gene Expression' OR Mutation OR 'Pathology, Molecular' OR 'Chromosome Aberrations' OR 'molecular diagnostics' OR 'molecular markers' OR 'molecular alterations' OR 'genetic alterations' OR 'genomic analysis' OR 'molecular profiling') **AND** (Mouth OR Jaw OR Maxillofacial OR 'Head and Neck' OR 'oral and maxillofacial' OR jaws OR mandible OR maxilla OR craniofacial OR oral OR 'oral cavity' OR extraskeletal) | 1082 |
| **Web of Science** | TS=(Osteosarcoma OR Osteosarcomas OR "Osteogenic sarcoma" OR "osteosarcoma tumor" OR "osteosarcoma tumors" OR "Tumor, Osteosarcoma" OR "Tumors, Osteosarcoma" OR "Sarcoma, Osteogenic" OR "Osteogenic Sarcomas" OR "Sarcomas, Osteogenic" OR “bone tumours” OR “bone tumor" OR “Neoplasms, bone tissue") **AND** TS=("Gene Expression" OR "Mutation" OR "Pathology, Molecular" OR "Chromosome Aberrations" OR "molecular diagnostics" OR "molecular markers" OR "molecular alterations" OR "genetic alterations" OR "genomic analysis" OR "molecular profiling") **AND** TS=(Mouth OR Jaw OR Maxillofacial OR "Head and Neck" OR "oral and maxillofacial" OR jaws OR mandible OR maxilla OR craniofacial OR oral OR "oral cavity" OR extraskeletal) | 108 |
| **Grey Literature** | | |
| **Google Scholar** | First 100 more relevant hits. No patents and no citations.  (osteosarcoma OR “osteogenic sarcoma”) **AND** (“molecular diagnostic” OR “molecular markers” OR “molecular analysis”) **AND** ("head and neck" OR "oral and maxillofacial") | 100 |
| **ProQuest** | TI,AB(osteosarcoma OR “osteogenic sarcoma”) **AND** TI,AB (“molecular diagnostic” OR “molecular markers” OR “molecular analysis”)  **AND** TI,AB("head and neck" OR "oral and maxillofacial") | 140 |
| **Total** |  | **2496** |

**Figure S1** Flow diagram of literature search and selection process adapted from PRISMA.


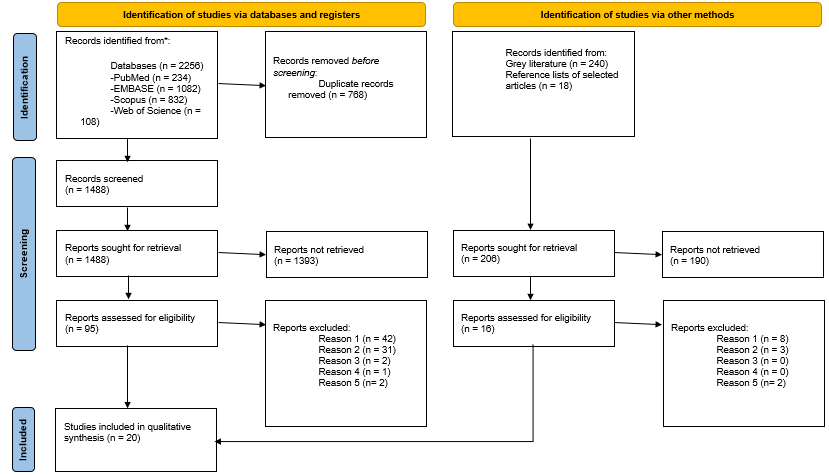


**Table S2** Excluded articles in Phase 2 and the respective reasons for their exclusion (n=91).

| References | Reasons for exclusion |
| --- | --- |
| 1. Grünewald TG, Alonso M, Avnet S, Banito A, Burdach S, Cidre-Aranaz F, Di Pompo G, Distel M, Dorado-Garcia H, Garcia-Castro J, González-González L, Grigoriadis AE, Kasan M, Koelsche C, Krumbholz M, Lecanda F, Lemma S, Longo DL, Madrigal-Esquivel C, Morales-Molina Á, Musa J, Ohmura S, Ory B, Pereira-Silva M, Perut F, Rodriguez R, Seeling C, Al Shaaili N, Shaabani S, Shiavone K, Sinha S, Tomazou EM, Trautmann M, Vela M, Versleijen-Jonkers YM, Visgauss J, Zalacain M, Schober SJ, Lissat A, English WR, Baldini N, Heymann D. Sarcoma treatment in the era of molecular medicine. EMBO Mol Med. 2020 Nov 6;12(11):e11131. doi: 10.15252/emmm.201911131. Epub 2020 Oct 13. PMID: 33047515; PMCID: PMC7645378. | 2 |
| 1. Cleven AHG, Schreuder WH, Groen E, Kroon HM, Baumhoer D. Molecular findings in maxillofacial bone tumours and its diagnostic value. Virchows Arch. 2020 Jan;476(1):159-174. doi: 10.1007/s00428-019-02726-2. Epub 2019 Dec 14. PMID: 31838586; PMCID: PMC6968989. | 2 |
| 1. Haefliger S, Andrei V, Baumhoer D. Update of Key Clinical, Histological and Molecular Features of Malignant Bone Tumours Arising in the Craniofacial Skeleton. Front Oncol. 2022 Jul 7;12:954717. doi: 10.3389/fonc.2022.954717. PMID: 35875137; PMCID: PMC9301068. | 2 |
| 1. Xu B. Proceedings of the North American Society of Head and Neck Pathology, Los Angeles, CA, March 20, 2022. Emerging Bone and Soft Tissue Neoplasms in the Head and Neck Region. Head Neck Pathol. 2022 Mar;16(1):158-167. doi: 10.1007/s12105-022-01418-9. Epub 2022 Mar 21. PMID: 35307771; PMCID: PMC9019004. | 1 |
| 1. Kumar P, Surya V, Urs AB, Augustine J, Mohanty S, Gupta S. Sarcomas of the Oral and Maxillofacial Region: Analysis of 26 Cases with Emphasis on Diagnostic Challenges. Pathol Oncol Res. 2019 Apr;25(2):593-601. doi: 10.1007/s12253-018-0510-9. Epub 2018 Oct 31. PMID: 30382526. | 1 |
| 1. Berner K, Bjerkehagen B, Bruland ØS, Berner A. Extraskeletal osteosarcoma in Norway, between 1975 and 2009, and a brief review of the literature. Anticancer Res. 2015 Apr;35(4):2129-40. PMID: 25862869. | 1 |
| 1. Machado I, Llombart-Bosch A, Charville GW, Navarro S, Domínguez Franjo MP, Bridge JA, Linos K. Sarcomas with EWSR1::Non-ETS Fusion (EWSR1::NFATC2 and EWSR1::PATZ1). Surg Pathol Clin. 2024 Mar;17(1):31-55. doi: 10.1016/j.path.2023.07.001. Epub 2023 Aug 7. PMID: 38278606. | 1 |
| 1. Fisch, A. S., St. John, M. A., & Sajed, D. P. (2023). Molecular Pathology of Head and Neck Tumors. In *Molecular Surgical Pathology* (pp. 493-516). Cham: Springer International Publishing. | 2 |
| 1. Tabareau-Delalande, F., de Pinieux, G. Marcadores moleculares de lesões fibro-ósseas e osteossarcomas do complexo craniofacial — situação atual e avanços recentes. *Curr Oral Health Rep*3 , 102–110 (2016). https://doi.org/10.1007/s40496-016-0086-y | 2 |
| 1. Romeo, S., Dei Tos, A. P., & Hogendoorn, P. C. (2012). The clinical impact of molecular techniques on diagnostic pathology of soft tissue and bone tumours. *Diagnostic Histopathology*, *18*(2), 81-85. | 2 |
| 1. Judson I. Targeted therapies in soft tissue sarcomas. Ann Oncol. 2010 Oct;21 Suppl 7:vii277-80. doi: 10.1093/annonc/mdq288. PMID: 20943628. | 1 |
| 1. van den Berg, H. (2006). Biology and therapy of solid tumors in childhood. *Update on Cancer Therapeutics*, *1*(3), 367-383. | 2 |
| 1. Patnaik S, Nayak BK, Das BR. Rearrangement in the coding and 5' region of p53 gene in human oral tumors. IUBMB Life. 1999 Sep;48(3):305-9. doi: 10.1080/713803524. PMID: 10690643. | 1 |
| 1. Woo VL, Pharar J, Herschaft EE, Hughes CC, Akerson HA, Danforth RA. Facial asymmetry associated with a mixed radiolucent-radiopaque change of the maxillofacial bones. J Am Dent Assoc. 2014 Mar;145(3):274-9. doi: 10.14219/jada.2013.42. PMID: 24583893. | 1 |
| 1. Hameed M, Horvai AE, Jordan RCK. Soft Tissue Special Issue: Gnathic Fibro-Osseous Lesions and Osteosarcoma. Head Neck Pathol. 2020 Mar;14(1):70-82. doi: 10.1007/s12105-019-01094-2. Epub 2020 Jan 16. PMID: 31950477; PMCID: PMC7021863. | 2 |
| 1. Bertin, H., Guilho, R., Brion, R., Amiaud, J., Battaglia, S., Moreau, A., ... & Rédini, F. (2019). Jaw osteosarcoma models in mice: first description. *Journal of Translational Medicine*, *17*, 1-8. | 2 |
| 1. de Alava, E. (2007). Molecular pathology in sarcomas. *Clinical and Translational Oncology*, *9*, 130-144. | 2 |
| 1. Gonin-Laurent N, Hadj-Hamou NS, Vogt N, Houdayer C, Gauthiers-Villars M, Dehainault C, Sastre-Garau X, Chevillard S, Malfoy B. RB1 and TP53 pathways in radiation-induced sarcomas. Oncogene. 2007 Sep 6;26(41):6106-12. doi: 10.1038/sj.onc.1210404. Epub 2007 Mar 19. PMID: 17369843. | 1 |
| 1. Tang N, Deng W, Wu Y, Deng Z, Wu X, Xiong J, Zhao Q. Decoding the role of SLC25A5 in osteosarcoma drug resistance and CD8+ T cell exhaustion: The therapeutic potential of phyllanthin. Phytomedicine. 2025 Jan;136:156291. doi: 10.1016/j.phymed.2024.156291. Epub 2024 Dec 8. PMID: 39752785. | 2 |
| 1. Liu, W., Shi, T., Zheng, D. *et al.* Identificação do fator inflamatório-1 do aloenxerto suprimindo a progressão e indicando bom prognóstico do osteossarcoma. *BMC Musculoskelet Disord*25 , 233 (2024). https://doi.org/10.1186/s12891-024-07363-8 | 2 |
| 1. Yao Y, Zhang Q, Li Z, Zhang H. MDM2: current research status and prospects of tumor treatment. Cancer Cell Int. 2024 May 13;24(1):170. doi: 10.1186/s12935-024-03356-8. PMID: 38741108; PMCID: PMC11092046. | 2 |
| 1. Macy ME, Mody R, Reid JM, Piao J, Saguilig L, Alonzo TA, Berg SL, Fox E, Weigel BJ, Hawkins DS, Mooney MM, Williams PM, Patton DR, Coffey BD, Roy-Chowdhuri S, Takebe N, Tricoli JV, Janeway KA, Seibel NL, Parsons DW. Palbociclib in Solid Tumor Patients With Genomic Alterations in the cyclinD-cdk4/6-INK4a-Rb Pathway: Results From National Cancer Institute-Children's Oncology Group Pediatric Molecular Analysis for Therapy Choice Trial Arm I (APEC1621I). JCO Precis Oncol. 2024 Sep;8:e2400418. doi: 10.1200/PO-24-00418. PMID: 39298716; PMCID: PMC11488755. | 2 |
| 1. Fan J, Liao J, Huang Y. Combined bioinformatics and machine learning methodologies reveal prognosis-related ceRNA network and propose ABCA8, CAT, and CXCL12 as independent protective factors against osteosarcoma. Adv Clin Exp Med. 2024 Aug;33(8):857-868. doi: 10.17219/acem/172663. PMID: 38315381. | 1 |
| 1. Nie J, He C, Shu Z, Liu N, Zhong Y, Long X, Liu J, Yang F, Liu Z, Huang P. Identification and experimental validation of Stearoyl-CoA desaturase is a new drug therapeutic target for osteosarcoma. Eur J Pharmacol. 2024 Jan 15;963:176249. doi: 10.1016/j.ejphar.2023.176249. Epub 2023 Dec 7. PMID: 38070637. | 2 |
| 1. Gu Y, Song Y, Pan Y, Liu J. The essential roles of m^6^A modification in osteogenesis and common bone diseases. Genes Dis. 2023 Mar 28;11(1):335-345. doi: 10.1016/j.gendis.2023.01.032. PMID: 37588215; PMCID: PMC10425797. | 2 |
| 1. Li A, Hancock JC, Quezado M, Ahn S, Briceno N, Celiku O, Ranjan S, Aboud O, Colwell N, Kim SA, Nduom E, Kuhn S, Park DM, Vera E, Aldape K, Armstrong TS, Gilbert MR. TGF-β and BMP signaling are associated with the transformation of glioblastoma to gliosarcoma and then osteosarcoma. Neurooncol Adv. 2023 Dec 19;6(1):vdad164. doi: 10.1093/noajnl/vdad164. PMID: 38292240; PMCID: PMC10825841. | 2 |
| 1. Wang, J., Zheng, L., Chen, W. *et al.* O direcionamento de RBM39 suprime o crescimento tumoral e sensibiliza as células do osteossarcoma à cisplatina. *Oncogene* (2024). https://doi.org/10.1038/s41388-024-03242-7 | 2 |
| 1. Pepper, T., Carey, B., Alibhai, M., Fry, A., & Sandison, A. (2023). 24. Sarcomas presenting as epithelial neoplasia in the head and neck-a case series. *British Journal of Oral and Maxillofacial Surgery*, *61*(10), e9-e10. | 2 |
| 1. Filippini DM, Carosi F, Querzoli G, Fermi M, Ricciotti I, Molteni G, Presutti L, Foschini MP, Locati LD. Rare Head and Neck Cancers and Pathological Diagnosis Challenges: A Comprehensive Literature Review. Diagnostics (Basel). 2024 Oct 23;14(21):2365. doi: 10.3390/diagnostics14212365. PMID: 39518333; PMCID: PMC11544949. | 2 |
| 1. Sombutthaweesri T, Wu S, Chamusri N, Settakorn J, Pruksakorn D, Chaiyawat P, Sastraruji T, Krisanaprakornkit S, Supanchart C. Relationship Between O-GlcNAcase Expression and Prognosis of Patients With Osteosarcoma. Appl Immunohistochem Mol Morphol. 2022 Jan 1;30(1):e1-e10. doi: 10.1097/PAI.0000000000000970. PMID: 34469899. | 1 |
| 1. Sonaglio V, de Carvalho AC, Toledo SR, Salinas-Souza C, Carvalho AL, Petrilli AS, de Camargo B, Vettore AL. Aberrant DNA methylation of ESR1 and p14ARF genes could be useful as prognostic indicators in osteosarcoma. Onco Targets Ther. 2013 Jun 17;6:713-23. doi: 10.2147/OTT.S44918. PMID: 23836983; PMCID: PMC3699305. | 1 |
| 1. Weber V, Stigler R, Lutz R, Kesting M, Weber M. Systematic review of craniofacial osteosarcoma regarding different clinical, therapeutic and prognostic parameters. Front Oncol. 2023 Mar 24;13:1006622. doi: 10.3389/fonc.2023.1006622. PMID: 37035145; PMCID: PMC10080080. | 1 |
| 1. Yan GN, Lv YF, Guo QN. Advances in osteosarcoma stem cell research and opportunities for novel therapeutic targets. Cancer Lett. 2016 Jan 28;370(2):268-74. doi: 10.1016/j.canlet.2015.11.003. Epub 2015 Nov 10. PMID: 26571463. | 2 |
| 1. Pakos EE, Kyzas PA, Ioannidis JP. Prognostic significance of TP53 tumor suppressor gene expression and mutations in human osteosarcoma: a meta-analysis. Clin Cancer Res. 2004 Sep 15;10(18 Pt 1):6208-14. doi: 10.1158/1078-0432.CCR-04-0246. PMID: 15448009. | 1 |
| 1. Amer HW, Algadi HH, Hamza SA. Mandibular small cell osteosarcoma: a case report and review of literature. J Egypt Natl Canc Inst. 2023 Sep 18;35(1):30. doi: 10.1186/s43046-023-00191-2. PMID: 37718329. | 1 |
| 1. Li Z, Dou P, Liu T, He S. Application of Long Noncoding RNAs in Osteosarcoma: Biomarkers and Therapeutic Targets. Cell Physiol Biochem. 2017;42(4):1407-1419. doi: 10.1159/000479205. Epub 2017 Jul 17. PMID: 28715796. | 2 |
| 1. Varachev V, Shekhtman A, Guskov D, Rogozhin D, Zasedatelev A, Nasedkina T. Diagnostics of *IDH1/2* Mutations in Intracranial Chondroid Tumors: Comparison of Molecular Genetic Methods and Immunohistochemistry. Diagnostics (Basel). 2024 Jan 16;14(2):200. doi: 10.3390/diagnostics14020200. PMID: 38248076; PMCID: PMC10814347. | 2 |
| 1. Wu CC, Beird HC, Andrew Livingston J, Advani S, Mitra A, Cao S, Reuben A, Ingram D, Wang WL, Ju Z, Hong Leung C, Lin H, Zheng Y, Roszik J, Wang W, Patel S, Benjamin RS, Somaiah N, Conley AP, Mills GB, Hwu P, Gorlick R, Lazar A, Daw NC, Lewis V, Futreal PA. Immuno-genomic landscape of osteosarcoma. Nat Commun. 2020 Feb 21;11(1):1008. doi: 10.1038/s41467-020-14646-w. PMID: 32081846; PMCID: PMC7035358. | 1 |
| 1. Yu XW, Wu TY, Yi X, Ren WP, Zhou ZB, Sun YQ, Zhang CQ. Prognostic significance of VEGF expression in osteosarcoma: a meta-analysis. Tumour Biol. 2014 Jan;35(1):155-60. doi: 10.1007/s13277-013-1019-1. Epub 2013 Aug 2. PMID: 23907576. | 1 |
| 1. Jiang L, Tao C, He A. Prognostic significance of p53 expression in malignant bone tumors: a meta-analysis. Tumour Biol. 2013 Apr;34(2):1037-43. doi: 10.1007/s13277-012-0643-5. Epub 2013 Jan 23. PMID: 23341181. | 1 |
| 1. Fukunaga M. Low-grade central osteosarcoma of the skull. Pathol Res Pract. 2005;201(2):131-5. doi: 10.1016/j.prp.2004.12.001. PMID: 15901134. | 1 |
| 1. Deshpande, A. M. (2003). *Characterization of a novel tumor suppressor gene involved in osteosarcoma tumorigenesis*. University of Connecticut. | 1 |
| 1. Wang L, Liu Y, Yu G. Avasimibe inhibits tumor growth by targeting FoxM1-AKR1C1 in osteosarcoma. Onco Targets Ther. 2019 Jan 24;12:815-823. doi: 10.2147/OTT.S165647. PMID: 30774369; PMCID: PMC6353227. | 1 |
| 1. Miyashita, H., Yoshida, K., Kameyama, K., Yazawa, M., Nakagawa, T., & Kawana, H. (2017). A case of dedifferentiated parosteal osteosarcoma in the maxilla. *International Journal of Oral and Maxillofacial Surgery*, *46*, 291. | 1 |
| 1. Fu D, Lu C, Qu X, Li P, Chen K, Shan L, Zhu X. LncRNA TTN-AS1 regulates osteosarcoma cell apoptosis and drug resistance via the miR-134-5p/MBTD1 axis. Aging (Albany NY). 2019 Oct 10;11(19):8374-8385. doi: 10.18632/aging.102325. Epub 2019 Oct 10. PMID: 31600142; PMCID: PMC6814585. | 1 |
| 1. Zhou, D., Wang, W., Yan, S., Ye, S., Wu, Y., Ding, Y., ... & Zhang, S. (2020). Comprehensive profiling of MDM2/TP53 genomic aberration in Chinese patients with diverse malignancies. | 2 |
| 1. Rattanakuntee, S., Chaiyawat, P., Pruksakorn, D., Kritsanaprakornkit, S., Makeudom, A., & Supanchart, C. (2020). Associations between Expression Levels of O-GlcNAc Transferase (OGT) and Chemo-Response in Osteosarcoma. *Journal of the Medical Association of Thailand*, *103*(2). | 3 |
| 1. Ege B, Yumrutas O, Ege M, Pehlivan M, Bozgeyik I. Pharmacological properties and therapeutic potential of saffron (Crocus sativus L.) in osteosarcoma. J Pharm Pharmacol. 2020 Jan;72(1):56-67. doi: 10.1111/jphp.13179. Epub 2019 Oct 23. PMID: 31645086. | 1 |
| 1. Chopra, S. (2020, March). SATB2 Positivity Not Specific for Osteoblastic Differentiation and Caution Needed in Making Diagnoses of Osteosarcoma in the Absence of Histological or Radiological Evidence of Osteoid. In *LABORATORY INVESTIGATION* (Vol. 100, No. SUPPL 1, pp. 39-39). 75 VARICK ST, 9TH FLR, NEW YORK, NY 10013-1917 USA: NATURE PUBLISHING GROUP. | 2 |
| 1. Liu JY, Zhu BR, Wang YD, Sun X. The efficacy and safety of Apatinib mesylate in the treatment of metastatic osteosarcoma patients who progressed after standard therapy and the VEGFR2 gene polymorphism analysis. Int J Clin Oncol. 2020 Jun;25(6):1195-1205. doi: 10.1007/s10147-020-01644-7. Epub 2020 Mar 25. PMID: 32215805. | 1 |
| 1. Liu JF, Lee CW, Lin CY, Chao CC, Chang TM, Han CK, Huang YL, Fong YC, Tang CH. CXCL13/CXCR5 Interaction Facilitates VCAM-1-Dependent Migration in Human Osteosarcoma. Int J Mol Sci. 2020 Aug 24;21(17):6095. doi: 10.3390/ijms21176095. PMID: 32847038; PMCID: PMC7504668. | 1 |
| 1. Liu JF, Chen PC, Chang TM, Hou CH. Thrombospondin-2 stimulates MMP-9 production and promotes osteosarcoma metastasis via the PLC, PKC, c-Src and NF-κB activation. J Cell Mol Med. 2020 Nov;24(21):12826-12839. doi: 10.1111/jcmm.15874. Epub 2020 Oct 6. PMID: 33021341; PMCID: PMC7686970. | 1 |
| 1. Chen D, Wan B, Cheng Y, Luo Y, Bai X, Guo J, Li G, Jin T, Nie J, Liu W, Wang R. Carboxypeptidase E is a prognostic biomarker co-expressed with osteoblastic genes in osteosarcoma. PeerJ. 2023 Aug 30;11:e15814. doi: 10.7717/peerj.15814. PMID: 37663298; PMCID: PMC10474831. | 1 |
| 1. Zhang Z, Zhang J, Duan Y, Li X, Pan J, Wang G, Shen B. Identification of B cell marker genes based on single-cell sequencing to establish a prognostic model and identify immune infiltration in osteosarcoma. Front Immunol. 2022 Dec 7;13:1026701. doi: 10.3389/fimmu.2022.1026701. PMID: 36569871; PMCID: PMC9774034. | 2 |
| 1. Arman K, Saadat KASM, Igci YZ, Bozgeyik E, Ikeda MA, Cakmak EA, Arslan A. Long noncoding RNA ERICD interacts with ARID3A via E2F1 and regulates migration and proliferation of osteosarcoma cells. Cell Biol Int. 2020 Nov;44(11):2263-2274. doi: 10.1002/cbin.11434. Epub 2020 Aug 10. PMID: 32749762. | 2 |
| 1. Arman K, Saadat KASM, Igci YZ, Bozgeyik E, Ikeda MA, Cakmak EA, Arslan A. Long noncoding RNA ERICD interacts with ARID3A via E2F1 and regulates migration and proliferation of osteosarcoma cells. Cell Biol Int. 2020 Nov;44(11):2263-2274. doi: 10.1002/cbin.11434. Epub 2020 Aug 10. PMID: 32749762. | 1 |
| 1. Yi GZ, Zhu TC, Que TS, Li ZY, Huang GL. Individualized combination therapies based on whole-exome sequencing displayed significant clinical benefits in a glioblastoma patient with secondary osteosarcoma: case report and genetic characterization. BMC Neurol. 2022 Oct 21;22(1):390. doi: 10.1186/s12883-022-02920-x. PMID: 36271359; PMCID: PMC9587562. | 1 |
| 1. Jiang Y, Wang J, Sun M, Zuo D, Wang H, Shen J, Jiang W, Mu H, Ma X, Yin F, Lin J, Wang C, Yu S, Jiang L, Lv G, Liu F, Xue L, Tian K, Wang G, Zhou Z, Lv Y, Wang Z, Zhang T, Xu J, Yang L, Zhao K, Sun W, Tang Y, Cai Z, Wang S, Hua Y. Multi-omics analysis identifies osteosarcoma subtypes with distinct prognosis indicating stratified treatment. Nat Commun. 2022 Nov 23;13(1):7207. doi: 10.1038/s41467-022-34689-5. PMID: 36418292; PMCID: PMC9684515. | 1 |
| 1. Zheng W, Li S, Huang J, Dong Y, Zhang H, Zheng J. Down-Regulation of Ubiquitin-Specific Peptidase 9X Inhibited Proliferation, Migration and Invasion of Osteosarcoma via ERK1/2 and PI3K/Akt Signaling Pathways. Biol Pharm Bull. 2022;45(9):1283-1290. doi: 10.1248/bpb.b22-00198. PMID: 36047196. | 1 |
| 1. Hong J, Li Q, Wang X, Li J, Ding W, Hu H, He L. Development and validation of apoptosis-related signature and molecular subtype to improve prognosis prediction in osteosarcoma patients. J Clin Lab Anal. 2022 Jul;36(7):e24501. doi: 10.1002/jcla.24501. Epub 2022 May 16. PMID: 35576501; PMCID: PMC9280000. | 1 |
| 1. Mai W, Kong L, Yu H, Bao J, Song C, Qu G. Glycogen synthase kinase 3β promotes osteosarcoma invasion and migration via regulating PTEN and phosphorylation of focal adhesion kinase. Biosci Rep. 2021 Jul 30;41(7):BSR20193514. doi: 10.1042/BSR20193514. PMID: 33969873; PMCID: PMC8314432. | 1 |
| 1. Luo T, Zhou X, Jiang E, Wang L, Ji Y, Shang Z. Osteosarcoma Cell-Derived Small Extracellular Vesicles Enhance Osteoclastogenesis and Bone Resorption Through Transferring MicroRNA-19a-3p. Front Oncol. 2021 Mar 25;11:618662. doi: 10.3389/fonc.2021.618662. PMID: 33842319; PMCID: PMC8029976. | 2 |
| 1. Conley AP, Trent J, Zhang W. Recent progress in the genomics of soft tissue sarcomas. Curr Opin Oncol. 2008 Jul;20(4):395-9. doi: 10.1097/CCO.0b013e328302edc0. PMID: 18525334. | 1 |
| 1. Ordóñez JL, Osuna D, García-Domínguez DJ, Amaral AT, Otero-Motta AP, Mackintosh C, Sevillano MV, Barbado MV, Hernández T, de Alava E. The clinical relevance of molecular genetics in soft tissue sarcomas. Adv Anat Pathol. 2010 May;17(3):162-81. doi: 10.1097/PAP.0b013e3181d98cbf. PMID: 20418671. | 1 |
| 1. VAYEGO, S. A., DE CONTI, O. J., & SILVA, A. E. Aspectos genético-moleculares dos tumores ósseos. | 2 |
| 1. Donner, L. R. (1994). Cytogenetics of tumors of soft tissue and bone: implication for pathology. *Cancer genetics and cytogenetics*, *78*(2), 115-126. | 2 |
| 1. Dai Z, Liu Z, Yang R, Cao W, Ji T. EVI2B Is a Prognostic Biomarker and Is Correlated with Monocyte and Macrophage Infiltration in Osteosarcoma Based on an Integrative Analysis. Biomolecules. 2023 Feb 8;13(2):327. doi: 10.3390/biom13020327. PMID: 36830696; PMCID: PMC9953216. | 1 |
| 1. de Álava, E. Molecular pathology in sarcomas. *Clin Transl Oncol* 9, 130–144 (2007). https://doi.org/10.1007/s12094-007-0027-2 | 2 |
| 1. Deutsch EC, Seyer LA, Perlman SL, Yu J, Lynch DR. Clinical monitoring in a patient with Friedreich ataxia and osteogenic sarcoma. J Child Neurol. 2012 Sep;27(9):1159-63. doi: 10.1177/0883073812448460. Epub 2012 Jun 29. PMID: 22752483; PMCID: PMC3674811. | 2 |
| 1. Salaün, H., Le Nail, L. R., Simon, C., Narciso, B., De Pinieux, G., Vegas, H., & Vinceneux, A. (2022). Unexpected severe hepatic and skin toxicities during high dose methotrexate course for osteosarcoma. *Journal of Oncology Pharmacy Practice*, *28*(6), 1458-1464. | 1 |
| 1. Persha HE, Kato S, De P, Adashek JJ, Sicklick JK, Subbiah V, Kurzrock R. Osteosarcoma with cell-cycle and fibroblast growth factor genomic alterations: case report of Molecular Tumor Board combination strategy resulting in long-term exceptional response. J Hematol Oncol. 2022 Aug 28;15(1):119. doi: 10.1186/s13045-022-01344-x. PMID: 36031605; PMCID: PMC9420268. | 1 |
| 1. de Maeyer VM, Kestelyn PA, Shah AD, Van Den Broecke CM, Denys HG, Decock CE. Extraskeletal osteosarcoma of the orbit: A clinicopathologic case report and review of literature. Indian J Ophthalmol. 2016 Sep;64(9):687-689. doi: 10.4103/0301-4738.97555. PMID: 27853024; PMCID: PMC5151166. | 1 |
| 1. Lopes-Brás R, Lopez-Presa D, Esperança-Martins M, Melo-Alvim C, Gallego L, Costa L, Fernandes I. Genomic Profiling of Sarcomas: A Promising Weapon in the Therapeutic Arsenal. Int J Mol Sci. 2022 Nov 17;23(22):14227. doi: 10.3390/ijms232214227. PMID: 36430703; PMCID: PMC9693140. | 2 |
| 1. Wu P, Wu D, Zhao L, Huang L, Shen G, Huang J, Chai Y. Prognostic role of STAT3 in solid tumors: a systematic review and meta-analysis. Oncotarget. 2016 Apr 12;7(15):19863-83. doi: 10.18632/oncotarget.7887. PMID: 26959884; PMCID: PMC4991424. | 1 |
| 1. Pan L, Meng L, Liang F, Cao L. miR‑188 suppresses tumor progression by targeting SOX4 in pediatric osteosarcoma. Mol Med Rep. 2018 Jul;18(1):441-446. doi: 10.3892/mmr.2018.8997. Epub 2018 May 9. PMID: 29749512. | 1 |
| 1. Bertin H, Gomez-Brouchet A, Rédini F. Osteosarcoma of the jaws: An overview of the pathophysiological mechanisms. Crit Rev Oncol Hematol. 2020 Dec;156:103126. doi: 10.1016/j.critrevonc.2020.103126. Epub 2020 Oct 10. PMID: 33113487. | 2 |
| 1. Zhu, G., Lu, C., Nafa, K., Klein, M., Hwang, S., Wang, L., & Hameed, M. (2019, March). Comprehensive genomic profiling of the primary craniofacial osteosarcomas. In *LABORATORY INVESTIGATION* (Vol. 99). 75 VARICK ST, 9TH FLR, NEW YORK, NY 10013-1917 USA: NATURE PUBLISHING GROUP. | 1 |
| 1. Junior AT, de Abreu Alves F, Pinto CA, Carvalho AL, Kowalski LP, Lopes MA. Clinicopathological and immunohistochemical analysis of twenty-five head and neck osteosarcomas. Oral Oncol. 2003 Jul;39(5):521-30. doi: 10.1016/s1368-8375(03)00017-4. PMID: 12747978. | 1 |
| 1. Si, Xh., Liu, Z. Expression of cyclin D1 and CDK4 in osteosarcoma of the jaws. *Chin. J. Cancer Res.* 13, 140–143 (2001). https://doi.org/10.1007/s11670-001-0033-9 | 1 |
| 1. Chen WL, Feng HJ, Li HG. Expression and significance of hypoxemia-inducible factor-1alpha in osteosarcoma of the jaws. Oral Surg Oral Med Oral Pathol Oral Radiol Endod. 2008 Aug;106(2):254-7. doi: 10.1016/j.tripleo.2008.01.029. Epub 2008 Jun 13. PMID: 18554951. | 1 |
| 1. Asioli S, Righi A, Rucci P, Tarsitano A, Marchetti C, Bacchini P, Balbi T, Bertoni F, Foschini MP. p16 protein expression and correlation with clinical and pathological features in osteosarcoma of the jaws: Experience of 37 cases. Head Neck. 2017 Sep;39(9):1825-1831. doi: 10.1002/hed.24835. Epub 2017 May 31. PMID: 28560748. | 1 |
| 1. Zelinka, J., Blahák, J., Daněk, Z., & Bulik, O. (2018). Chondroblastic osteosarcoma of maxilla, a patient with Li-Fraumeni syndrome. *Česká a Slovenská Neurologie a Neurochirurgie*, *81*. | 3 |
| 1. Wang L, Wu Q, Sun K. [Image analysis of cell nuclear morphology and DNA content in osteosarcoma of the jaws]. Zhonghua Kou Qiang Yi Xue Za Zhi. 1997 Mar;32(2):78-80. Chinese. PMID: 10677953. | 4 |
| 1. Jensen MR, Stoltze U, Hansen TVO, Bak M, Sehested A, Rechnitzer C, Mathiasen R, Scheie D, Larsen KB, Olsen TE, Muhic A, Skjøth-Rasmussen J, Rossing M, Schmiegelow K, Wadt K. 9p21.3 Microdeletion involving *CDKN2A/2B* in a young patient with multiple primary cancers and review of the literature. Cold Spring Harb Mol Case Stud. 2022 Jun 22;8(4):a006164. doi: 10.1101/mcs.a006164. PMID: 35422439; PMCID: PMC9235845. | 1 |
| 1. Wen B, Chen J, Ding T, Mao Z, Jin R, Wang Y, Shi M, Zhao L, Yang A, Qin X, Chen X. Development and experimental validation of hypoxia-related gene signatures for osteosarcoma diagnosis and prognosis based on WGCNA and machine learning. Sci Rep. 2024 Aug 12;14(1):18734. doi: 10.1038/s41598-024-69638-3. PMID: 39134603; PMCID: PMC11319349. | 1 |
| 1. McHugh, J. B., Thomas, D. G., Herman, J. M., Ray, M. E., Baker, L. H., Adsay, N. V., ... & Lucas, D. R. (2006). Primary versus radiation‐associated craniofacial osteosarcoma: biologic and clinicopathologic comparisons. *Cancer*, *107*(3), 554-562. | 5 |
| 1. Ji, J., Quindipan, C., Parham, D., Shen, L., Ruble, D., Bootwalla, M., ... & Mascarenhas, L. (2017). Inherited germline ATRX mutation in two brothers with ATR‐X syndrome and osteosarcoma. *American journal of medical genetics Part A*, *173*(5), 1390-1395. | 1 |
| 1. Kim, Y. D., Hwang, D. S., Kim, C. H., Shin, S. H., Kim, U. K., Kim, J. R., & Chung, I. K. (2007). Impact of Methylation of the Gene $ p16^{INK4a} $ on Prognosis of Head and Neck Osteosarcoma. *Journal of the Korean Association of Oral and Maxillofacial Surgeons*, *33*(1), 46-54. | 5 |
| 1. Jang, H. S., Cho, J. O., Yoon, C. Y., Kim, H. J., & Park, J. C. (2001). Demonstration of Epstein–Barr virus in odontogenic and nonodontogenic tumors by the polymerase chain reaction (PCR). *Journal of oral pathology & medicine*, *30*(10), 603-610. | 5 |
| 1. Ginat, D., Schulte, J., Gooi, Z., & Cipriani, N. (2018). High-grade conventional osteosarcoma of the mandible associated with P53 germline mutation. *Journal of Craniofacial Surgery*, *29*(3), 738-740. | 1 |
| 1. Wang, H., Rodgers, W. H., Chmell, M. J., Svitek, C., & Schwartz, H. S. (1995). Osteosarcoma oncogene expression detected by in situ hybridization. *Journal of orthopaedic research*, *13*(5), 671-678. | 5 |

1. Studies that did not address molecular alterations in oral and maxillofacial osteosarcoma;
2. Clinical trials, experimental studies, narrative reviews, protocols, brief communications, personal opinions, letters, book chapters, and conference abstracts;
3. Studies without full-text availability;
4. Studies published in languages other than English, Spanish, or Portuguese;
5. Lack of sufficient data.

**Table S3** Demographic, clinicopathological, and molecular characteristics of the 20 studies (68 cases) of oral and maxillofacial osteosarcoma included in the scoping review.

| **Author (year)** | **Country** | **Type of Study** | **N** | **Age (years)** | **Sex** | **Risk factors** | **Genetic factors** | **Other history** | **Location** | **Size (cm)** | **Histology** | **Grade** | **LVI** | **NPI** | **Necrosis** | **T** | **N** | **M** | **Stage** | **Molecular test** | ***TP53*** | ***MDM2*** | ***CDK4*** | ***GNAS*** | ***SAS*** | **OTHERS** | **Treatment** | **Surgical margins** | **LR** | **Metastases** | **Follow-up (months)** | **Status** |
| --- | --- | --- | --- | --- | --- | --- | --- | --- | --- | --- | --- | --- | --- | --- | --- | --- | --- | --- | --- | --- | --- | --- | --- | --- | --- | --- | --- | --- | --- | --- | --- | --- |
| Entz-Werle et al. (2003) | France | Cohort | 1 | 9 | NI | NI | NI | NI | MD | NI | NI | H | NI | NI | NI | NI | NI | NI | NI | PCR | Normal status | NI | NI | NI | NI | RB1, D9S171 (9p21), D5S346 (5q21):Allelic imbalance (heterozygous) ; D5S492 (5q21): homozygous | Surgery+CT | NI | NI | NI | 7 | DOD |
| Tahir et al.  (2024) | USA | Case report | 1 | 81 | F | NI | NI | High incidence of cancer in her paternal relatives | MD | 5.0x4.0 | TG | I | Pos | NI | No | pT1 | pN0 | NI | IA | NGS | Missense and/or frame shift mutation | NI | NI | NI | NI | Missense and frameshift mutations: MUC4, MUC6, MUC17, MUC20, HLA, ZNF221, ZNF417, ZNF517, ZNF595, ZNF774, ZNF831, CYP2A7, CYP27C1, GADD45B, MSH4, TDG, MCM4, RBBP8NL, PER3, CDK11B, CDC27, and CCNE1 | Surgery | NI | NI | NI | NI | NI |
| Liu et al. (2017) | USA | Case report | 1 | 17 | F | NI | NI | NI | MD | NI | NI | NI | NI | NI | Yes | NI | NI | NI | NI | FISH | Amp | NI | NI | NI | NI | Primary tumor: PTEN loss, TP53 R342, C17orf39 amplification and RB1 splice site 539+1G>A, Recurrent tumor: TP53 R342 mutation and a KRAS amplification | Surgery+CT+RT | Pos | No | Yes (bone marrow) | 58 | DOD |
| Ogi et al.  (2020) | Japan | Case report | 1 | 17 | M | NI | LFS | History of cancer in his paternal relatives | MD | 8.5x5.3x7.4 | CD | H | NI | NI | NI | NI | NI | NI | NI | PCR | Amp | NI | NI | NI | NI | NI | NI | NI | NI | NI | 120 | NED |
| Patrikidou et al.  (2002) | UK | Case report | 1 | 26 | F | NI | LFS | Hermaphroditism at age 1, osteosarcoma of the maxilla at age 26, and Bowen's disease at age 31. History of cancer in her paternal relatives | MX | NI | NI | NI | NI | NI | NI | NI | NI | NI | NI | PCR | Amp | NI | NI | NI | NI | NI | NI | NI | NI | NI | NI | Alive |
| Diniz et al.  (2011) | Brazil | Cross- sectional | 2 | 48 | F | NI | NI | NI | NI | NI | NI | NI | NI | NI | NI | NI | NI | NI | NI | qPCR; RT-PCR | NI | NI | NI | NI | NI | (1) WWOX: aberrant transcripts with total or partial loss of exon length | NI | NI | NI | NI | NI | NI |
|  |  |  |  | 34 | F | NI | NI | NI | NI | NI | NI | NI | NI | NI | NI | NI | NI | NI | NI |  | NI | NI | NI | NI | NI |  | NI | NI | NI | NI | NI | NI |
| Zhu et al. (2024) | USA | Cohort | 15 | 19 | M | NI | NI | No | MD | 11 | OB | H | NI | NI | NI | NI | NI | NI | NI | NGS: MSK-IMPACT, SNP Array | (11) TP53: missense mutations (n = 8), frameshift truncation (n = 1), in-frame deletion (n = 1), and splice variant (n = 1). | NI | NI | Missense mutation R201C and R201H in two cases | NI | Other recurrently altered genes occurred at lower frequencies (2–3 cases /14), including CDKN2A/2B homozygous deletion, TERT amplification or hotspot mutation in the promoter, PBRM1 frameshift truncations, KMT2C missense mutations, APC missense mutations, PTEN homozygous deletion, frameshift truncation and missense mutation, RB1 homozygous deletion, missense mutation and in-frame deletion, and RAD50 missense mutation and in-frame fusion. RB1, TGFB1, and CBL were other mutated genes that occurred only in one case. | NI | NI | NI | NI | NI | NI |
|  |  |  |  | 58 | M | Post-radiation | NI | No | MX | 4.2 | OB CD | H | NI | NI | NI | NI | NI | NI | NI | NGS: MSK-IMPACT, SNP Array |  | NI | NI |  | NI |  | NI | NI | NI | NI | NI | NI |
|  |  |  |  | 67 | M | Post-radiation | NI | No | MD | 6.5 | OB CD FB | H | NI | NI | NI | NI | NI | NI | NI | NGS: MSK-IMPACT, SNP Array |  | NI | NI |  | NI |  | NI | NI | NI | NI | NI | NI |
|  |  |  |  | 54 | F | NI | NI | No | MX | 1.5 | OB | H | NI | NI | NI | NI | NI | NI | NI | NGS: MSK-IMPACT, SNP Array |  | NI | NI |  | NI |  | NI | NI | NI | NI | NI | NI |
|  |  |  |  | 54 | M | Post-radiation | NI | No | MD | 3.5 | OB CD FB | H | NI | NI | NI | NI | NI | NI | NI | NGS: MSK-IMPACT, SNP Array |  | NI | NI |  | NI |  | NI | NI | NI | NI | NI | NI |
|  |  |  |  | 22 | F | NI | NI | No | MD | 6.5 | OB CD | H | NI | NI | NI | NI | NI | NI | NI | NGS: MSK-IMPACT, SNP Array |  | NI | NI |  | NI |  | NI | NI | NI | NI | NI | NI |
|  |  |  |  | 52 | M | NI | NI | No | Frontal sinus  and infratemporal fossa | 7.0 | OB CD FB | H | NI | NI | NI | NI | NI | NI | NI | NGS: MSK-IMPACT, SNP Array |  | NI | NI |  | NI |  | NI | NI | NI | NI | NI | NI |
|  |  |  |  | 37 | F | NI | NI | No | MD | 5.3 | OB CD FB | H | NI | NI | NI | NI | NI | NI | NI | NGS: MSK-IMPACT, SNP Array |  | NI | NI |  | NI |  | NI | NI | NI | NI | NI | NI |
|  |  |  |  | 74 | F | NI | NI | No | MX | 6.5 | FB | H | NI | NI | NI | NI | NI | NI | NI | NGS: MSK-IMPACT, SNP Array |  | NI | NI |  | NI |  | NI | NI | NI | NI | NI | NI |
|  |  |  |  | 52 | M | Fibrous dysplasia | NI | No | MX | 4.0 | OB CD FB | H | NI | NI | NI | NI | NI | NI | NI | NGS: MSK-IMPACT, SNP Array |  | NI | NI |  | NI |  | NI | NI | NI | NI | NI | NI |
|  |  |  |  | 24 | M | NI | NI | No | MD | 8.0 | FB | H | NI | NI | NI | NI | NI | NI | NI | NGS: MSK-IMPACT, SNP Array |  | NI | NI |  | NI |  | NI | NI | NI | NI | NI | NI |
|  |  |  |  | 43 | M | NI | NI | No | MX | 4.5 | CD | H | NI | NI | NI | NI | NI | NI | NI | NGS: MSK-IMPACT, SNP Array |  | NI | NI |  | NI |  | NI | NI | NI | NI | NI | NI |
|  |  |  |  | 25 | M | NI | NI | No | MD | 5.5 | CD | H | NI | NI | NI | NI | NI | NI | NI | NGS: SNP Array |  | NI | NI |  | NI |  | NI | NI | NI | NI | NI | NI |
|  |  |  |  | 67 | M | NI | NI | No | MD | 5.3 | FB | H | NI | NI | NI | NI | NI | NI | NI | NGS: MSK-IMPACT, SNP Array |  | NI | NI |  | NI |  | NI | NI | NI | NI | NI | NI |
|  |  |  |  | 29 | F | NI | NI | No | Sphenoid | 2.9 | OB CD | H | NI | NI | NI | NI | NI | NI | NI | NGS: MSK-IMPACT, SNP Array |  | NI | NI |  | NI |  | NI | NI | NI | NI | NI | NI |
| Saucier et al.  (2024) | France | Cohort | 10 | 9 | NI | NI | LFS | No | MD | NI | CD | I | NI | NI | NI | NI | NI | NI | NI | NGS | Missense c.814G>A | NI | NI | NI | NI | NI | Surgery+CT | NI | No | No | 23 | Alive |
|  |  |  |  | 31.7 | NI | NI | LFS | 19.5y: 1st Osteosarcoma (limb). This is 2nd tumor, not metastasis | MD | NI | CD | I | NI | NI | NI | NI | NI | NI | NI | NGS | Missense c.842A>T | NI | NI | NI | NI | NI | Surgery | NI | Yes | No | 51 | Alive |
|  |  |  |  | 6.5 | NI | NI | LFS | No | MD | NI | OB CD | H | NI | NI | NI | NI | NI | NI | NI | NGS | Missense c.742C>T | NI | NI | NI | NI | NI | Surgery+CT | NI | Yes | No | 9 | Dead |
|  |  |  |  | 8.3 | NI | NI | LFS | 6.5y: 1st Osteosarcoma (jaw). This is the 2nd tumor, not metastasis | MD | NI | OB | H | NI | NI | NI | NI | NI | NI | NI | NGS | Missense c.742C>T | NI | NI | NI | NI | NI | Surgery+CT | NI | No | No | 9 | DOD |
|  |  |  |  | 12.8 | NI | NI | LFS | No | MD | NI | OB CD | H | NI | NI | NI | NI | NI | NI | NI | NGS | Missense c.518T>C | NI | NI | NI | NI | NI | Surgery+CT | NI | No | No | 16 | Alive |
|  |  |  |  | 19.2 | NI | NI | LFS | 11.9y: 1st Osteosarcoma (limb). This is the 2nd tumor, not metastasis | MD | NI | CD | H | NI | NI | NI | NI | NI | NI | NI | NGS | Missense c.844C>T | NI | NI | NI | NI | NI | Surgery+CT | NI | No | No | 24 | Alive |
|  |  |  |  | 15.7 | NI | NI | LFS | No | MD | NI | CD | H | NI | NI | NI | NI | NI | NI | NI | NGS | Missense c.717C>G | NI | NI | NI | NI | NI | Surgery+CT | NI | No | No | 16 | DOD |
|  |  |  |  | 20.9 | NI | NI | LFS | 16.7y: 1st Osteosarcoma (limb). This is 2nd tumor, not metastasis | MD | NI | CD | H | NI | NI | NI | NI | NI | NI | NI | NGS | Missence c.652_654del | NI | NI | NI | NI | NI | NI | NI | No | NI | 29 | Alive |
|  |  |  |  | 19 | NI | NI | LFS | 7y: 1st Osteosarcoma (axial). This is 2nd tumor, not metastasis | MD | NI | NI | H | NI | NI | NI | NI | NI | NI | NI | NGS | Missence c.833C>G | NI | NI | NI | NI | NI | Surgery+CT | NI | No | No | 24 | Alive |
|  |  |  |  | 19 | NI | NI | LFS | No | MD | NI | OB CD | H | NI | NI | NI | NI | NI | NI | NI | NGS | Missence  c.818G>A | NI | NI | NI | NI | NI | Surgery | NI | Yes | No | 24 | Alive |
| Garcia et al. (1990) | Spain | case report | 1 | 40 | F | Post-radiation: Acerebral astrocytoma | LFS | Lobular carcinoma of the breast with lymphatic involvement | MX | NI | NI | NI | NI | NI | NI | NI | NI | NI | NI | Single-strand conformation polymorphism analysis | G-+-A substitution at base 743 (codon 248, exon 7) of thep53 gene. | NI | NI | NI | NI | NI | Surgery | NI | No | Yes (multiple osseous locations) | 2 | DOD |
| Akouchekian et al.  (2016) | Iran | Case report | 1 | 43 | M | No | LFS | History of cancer in his paternal relatives | MD | NI | NI | NI | NI | NI | NI | NI | NI | NI | NI | PCR, DNA Sequencing, Multiplex ligation-dependent probe amplification (MLPA) | Amp | NI | NI | NI | NI | PTEN amplification | NI | NI | NI | NI | NI | NI |
| Li et al.  (2016) | China | Case report | 1 | 41 | F | Post-radiation | LFS | 7y: Neuroblastoma and rhabdomyosarcoma (upper lip); 28y: Cystosarcoma phyllode (breast); 29y: Bronchioalveolar cell carcinoma (bronchio); 39y: Infitranting ductal carcinoma (breast) and thyroi papillary carcinoma (thyroid); 41y: Osteosarcoma (maxila) and gastric adenocarcinoma (stomach); 43y: osteosarcoma (sternum); 45y: Acute myeloid leukemia (blood) | MX | NI | NI | NI | NI | NI | NI | NI | NI | NI | NI | NGS: whole-genome sequencing (WGS) and whole-exome sequencing (WES) | Two missense mutations (rs1042522 and rs28934576) in TP53 | NI | NI | NI | NI | NI | Surgery+CT | NI | NI | Not confirm that the sternal spindle cell sarcoma was a primary or a metastasis from the maxillary osteosarcoma. | 45 | DOC |
| Lopes et al. (2001) | Brazil | Cross- sectional | 9 | 32 | F | (1) Post-radiation:papillary thyroid carcinoma Latency:11 years Total dose: NI | No | No | MX | 1x1 | CD | L | NI | NI | NI | NI | NI | NI | NI | PCR | NI | Neg | Amp | NI | Neg | NI | Surgery | Neg | No | No | 11 | Alive |
|  |  |  |  | 36 | F |  | No | No | MX | 5x4 | Parosteal | L | NI | NI | NI | NI | NI | NI | NI |  | NI | Neg | Neg | NI | Amp | NI | Surgery | Neg | No | No | 12 | Alive |
|  |  |  |  | 20 | F |  | No | No | MD | 2.5x1.7 | CD | H | NI | NI | NI | NI | NI | NI | NI |  | NI | Amp | Amp | NI | Amp | NI | Surgery+CT | Neg | No | No | 33 | Alive |
|  |  |  |  | 63 | M |  | No | No | MX | 1.5x1 | CD | I | NI | NI | NI | NI | NI | NI | NI |  | NI | Amp | Amp | NI | Amp | NI | Surgery | Neg | No | No | 19 | Alive |
|  |  |  |  | 40 | F |  | No | No | MD | 3x1.5 | CD | I | NI | NI | NI | NI | NI | NI | NI |  | NI | Neg | Neg | NI | Neg | NI | Surgery | Neg | No | No | 60 | Alive |
|  |  |  |  | 46 | M |  | No | No | MX | 4x2 | OB | H | NI | NI | NI | NI | NI | NI | NI |  | NI | Amp | Amp | NI | Amp | NI | Surgery+RT | Close | No | No | 21 | Alive |
|  |  |  |  | 50 | F |  | No | No | MD | 1.6x1.5 | CD | L | NI | NI | NI | NI | NI | NI | NI |  | NI | Amp | Neg | NI | Amp | NI | Surgery | Neg | No | No | 10 | Alive |
|  |  |  |  | 51 | M |  | No | No | MX | 2.4x2 | CD | L | NI | NI | NI | NI | NI | NI | NI |  | NI | Amp | Amp | NI | Amp | NI | Surgery | Close | No | No | 6 | Alive |
|  |  |  |  | 64 | F |  | No | No | MD | 4.5x3 | OB | H | NI | NI | NI | NI | NI | NI | NI |  | NI | Amp | Amp | NI | Neg | NI | Surgery | Neg | No | No | 5 | Alive |
| Limbach et al. (2020) | USA | Cross- sectional | 4 | 62 | M | No | No | Prior colon cancer status post resection | MD | NI | OB | H | NI | NI | NI | NI | NI | NI | NI | FISH | NI | Amp | NI | NI | NI | NI | CT+RT | NI | NI | NI | 11 | DOC |
|  |  |  |  | 34 | M | No | No | No | MD | NI | OB | L | NI | NI | NI | NI | NI | NI | NI |  | NI | Neg | NI | NI | NI | NI | NI | NI | NI | NI | NI | LFU |
|  |  |  |  | 41 | F | No | No | No | MX | NI | FB | L | NI | NI | NI | NI | NI | NI | NI |  | NI | Failed | NI | NI | NI | NI | RT | NI | No | NI | 5 | Alive |
|  |  |  |  | NI | M | No | No | No | MX | NI | CD | L | NI | NI | NI | NI | NI | NI | NI |  | NI | Neg | NI | NI | NI | NI | NI | NI | NI | NI | NI | LFU |
| Hirose et al.  (2017) | Japan | Case report | 1 | 64 | M | No | No | No | MX | 3.3x2.2 | Giant cell–rich | H | NI | NI | NI | NI | NI | NI | NI | FISH | NI | Amp | Amp | NI | NI | NI | NI | NI | NI | NI | NI | NI |
| Haefliger et al.  2021) | Switzerland | Case report | 1 | 30 | F | Cemento-osseous dysplasia | LFS | Bilateral breast cancer | MD | NI | OB | H | NI | NI | Yes | NI | NI | NI | NI | NGS and Microarray for copy number analysis and loss of heterozygosity | p.V173G TP53 | NI | NI | Neg | NI | Chromoplexy with subsequent amplifications of 5p, 8pter, 12, and 19p as well as deletions of 5q, 6, 7, 10p, 13, and 22 | Surgery+CT | Neg | No | No | 36 | NED |
| Yap et al.  (2021) | Australia | Case report | 1 | 21 | M | Fibrous dysplasia | No | No | MX | 3.1x2.4x2.2 | NI | H | NI | NI | NI | NI | NI | NI | NI | NGS and FISH | Separate point mutation in TP53 at codon 281 (Asp281Asn) | Neg | Neg | Missense mutation Arg201Cys | NI | NI | Surgery+CT | NI | No | No | 10 | NED |
| Yokoyama et al.  (2023) | Japan | Case report | 1 | 61 | M | Post-radiation: squamous cell carcinoma Latency: 13 years Total dose: 60Gy | No | No | MX | NI | NI | NI | NI | NI | NI | NI | NI | NI | NI | NGS | TP53 K321fs*15 mutation were detected | NI | NI | NI | NI | Amp: CCNE1, KEL, EZH2, XRCC2 Missense mutation: LTK S183F, BRCA2 S758C, ERBB3 R1118Q, KMT2A (MLL) G73E Truncation mutation: LTK W707* | Surgery+CT | NI | No | Yes (Multiple lung) | 8 | AWD |
| Khan et al.  (2024) | India | Case report | 1 | 17 | F | No | No | No | MD | 8x10x4 | TG | NI | NI | NI | Yes | NI | NI | NI | NI | FISH | NI | Neg | Neg | NI | NI | NI | Surgery+CT | Neg | No | No | 8 | NED |
| Guérin et al.  (2016) | France | Cross- sectional | 14 | 61 | M | NI | NI | NI | MD | NI | CD | H | NI | NI | NI | NI | NI | NI | NI | PCR | NI | Neg | NI | Neg | NI | RASAL1: Neg | NI | NI | NI | NI | NI | NI |
|  |  |  |  | 32 | M | NI | NI | NI | MD | NI | CD | H | NI | NI | NI | NI | NI | NI | NI |  | NI | Neg | NI | Neg | NI | RASAL1: Neg | NI | NI | NI | NI | NI | NI |
|  |  |  |  | 80 | F | NI | NI | NI | MD | NI | FB | H | NI | NI | NI | NI | NI | NI | NI |  | NI | Amp | NI | Neg | NI | RASAL1: Amp | NI | NI | NI | NI | NI | NI |
|  |  |  |  | 55 | M | NI | NI | NI | MD | NI | FB | H | NI | NI | NI | NI | NI | NI | NI |  | NI | Amp | NI | Neg | NI | RASAL1: Amp | NI | NI | NI | NI | NI | NI |
|  |  |  |  | NI | NI | NI | NI | NI | MD | NI | FB | H | NI | NI | NI | NI | NI | NI | NI |  | NI | Neg | NI | Neg | NI | RASAL1: Neg | NI | NI | NI | NI | NI | NI |
|  |  |  |  | 80 | F | NI | NI | NI | MD | NI | FB | H | NI | NI | NI | NI | NI | NI | NI |  | NI | Amp | NI | Neg | NI | RASAL1: Amp | NI | NI | NI | NI | NI | NI |
|  |  |  |  | 21 | F | NI | NI | NI | MD | NI | FB | H | NI | NI | NI | NI | NI | NI | NI |  | NI | Neg | NI | Neg | NI | RASAL1: Neg | NI | NI | NI | NI | NI | NI |
|  |  |  |  | 31 | F | NI | NI | NI | MD | NI | FB | H | NI | NI | NI | NI | NI | NI | NI |  | NI | Neg | NI | Neg | NI | RASAL1: Neg | NI | NI | NI | NI | NI | NI |
|  |  |  |  | 61 | F | NI | NI | NI | MD | NI | FB | H | NI | NI | NI | NI | NI | NI | NI |  | NI | Amp | NI | Neg | NI | RASAL1: Neg | NI | NI | NI | NI | NI | NI |
|  |  |  |  | 49 | M | NI | NI | NI | MD | NI | FB | H | NI | NI | NI | NI | NI | NI | NI |  | NI | Amp | NI | Neg | NI | RASAL1: Neg | NI | NI | NI | NI | NI | NI |
|  |  |  |  | 26 | M | NI | NI | NI | MD | NI | FB | H | NI | NI | NI | NI | NI | NI | NI |  | NI | Neg | NI | Neg | NI | RASAL1: Neg | NI | NI | NI | NI | NI | NI |
|  |  |  |  | 46 | M | NI | NI | NI | MD | NI | OB | H | NI | NI | NI | NI | NI | NI | NI |  | NI | Neg | NI | Neg | NI | RASAL1: Neg | NI | NI | NI | NI | NI | NI |
|  |  |  |  | 68 | F | NI | NI | NI | MD | NI | OB | H | NI | NI | NI | NI | NI | NI | NI |  | NI | Neg | NI | Neg | NI | RASAL1: Neg | NI | NI | NI | NI | NI | NI |
|  |  |  |  | 60 | M | Post-radiation | NI | NI | MD | NI | OB | H | NI | NI | NI | NI | NI | NI | NI |  | NI | Neg | NI | Neg | NI | RASAL1: Neg | NI | NI | NI | NI | NI | NI |
| Khayat et al.  (2004) | Canada | Case report | 1 | 7 | NI | NI | NI | Rhabdomyosarcoma of scapula and adrenocortical carcinoma | MD | 2x3 | NI | NI | NI | NI | NI | NI | NI | NI | NI | NGS | Testing for p53 mutation by DNA sequence analysis yielded a positive result of a CGT > CAT mutation at codon 273 (Arg > His) | NI | NI | NI | NI | NI | CT | NI | No | Yes (Liver) | 7 | DOD |

LR (Local recurrence); H (High); I (Intermediate); L (Low); MD (Mandible); MX (Maxilla); LFS (Li-Fraumeni syndrome); Neg (Negative); Pos (Positive); LVI (Lymphovascular invasion); NPI (Neural/perineural invasion); FB (Fibroblastic); CD (Chondroblastic); OB (Osteoblastic); TG (Telangiectatic); Ampl (Amplification); NI (Not-Informed)

**Figure S2** World map illustrating the distribution of the included studies.


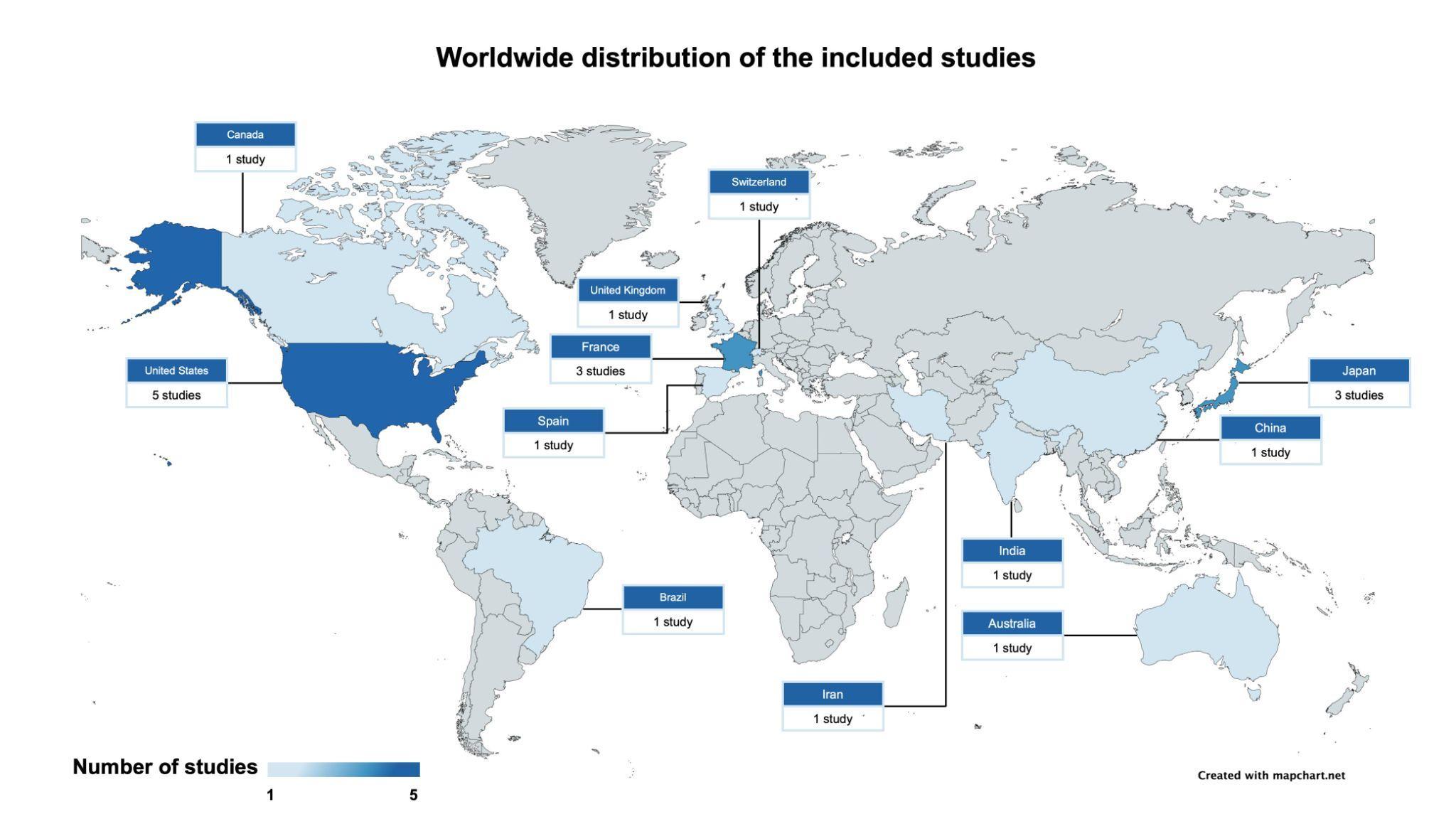

Supplement: Supplementary file 1 — Data S1: Supporting Information [file JOP-55-439-s001.docx]
